# A random subspace ensemble classification model for discrimination of power quality events in solar PV microgrid power network

**DOI:** 10.1371/journal.pone.0262570

**Published:** 2022-01-27

**Authors:** Arangarajan Vinayagam, Mohammad Lutfi Othman, Veerapandiyan Veerasamy, Suganthi Saravan Balaji, Kalaivani Ramaiyan, Padmavathi Radhakrishnan, Mohan Das Raman, Noor Izzri Abdul Wahab

**Affiliations:** 1 Department of Electrical and Electronics Engineering, New Horizon College of Engineering, Bangalore, India; 2 Advanced Lightning, Power and Energy Research (ALPER), Department of Electrical and Electronics Engineering, Universiti Putra Malaysia (UPM), Selangor, Malaysia; 3 School of Electrical and Electronic Engineering, Nanyang Technological University, Singapore, Singapore; 4 Department of Information Technology, College of Engineering and Computer Science, Lebanese French University, Erbil, Kurdistan Region, Iraq; 5 Department of Electrical and Electronics Engineering, Rajalakshmi Engineering College, Chennai, India; Hanyang University, REPUBLIC OF KOREA

## Abstract

This study proposes SVM based Random Subspace (RS) ensemble classifier to discriminate different Power Quality Events (PQEs) in a photovoltaic (PV) connected Microgrid (MG) model. The MG model is developed and simulated with the presence of different PQEs (voltage and harmonic related signals and distinctive transients) in both on-grid and off-grid modes of MG network, respectively. In the pre-stage of classification, the features are extracted from numerous PQE signals by Discrete Wavelet Transform (DWT) analysis, and the extracted features are used to learn the classifiers at the final stage. In this study, first three Kernel types of SVM classifiers (Linear, Quadratic, and Cubic) are used to predict the different PQEs. Among the results that Cubic kernel SVM classifier offers higher accuracy and better performance than other kernel types (Linear and Quadradic). Further, to enhance the accuracy of SVM classifiers, a SVM based RS ensemble model is proposed and its effectiveness is verified with the results of kernel based SVM classifiers under the standard test condition (STC) and varying solar irradiance of PV in real time. From the final results, it can be concluded that the proposed method is more robust and offers superior performance with higher accuracy of classification than kernel based SVM classifiers.

## 1. Introduction

Microgrid (MG) generally provides reliable, economic, and secured energy supply to the critical loads and remote areas of communities, with following additional features: promotes demand side management; low carbon emission of energy supply; accommodates multiple generating options from different types of Distributed Generation (DG) sources, and so on [1]. It is a major challenge to maintain the quality of energy supply in the MG network while penetration of nonlinear and unbalanced loads, renewable energy (RE) sources, and switching of heavy loads, capacitor banks, and network faults, etc. The PQEs (voltage sag, swell, harmonics, transients, interruptions, unbalanced voltage and current, etc.) can influence the overall performance of MG operation and shorten the life time of power devices integrated into the MG system [2, 3]. Therefore, to achieve improved PQ with safe and reliable operation of the MG power system, the source of PQEs must be detected and classified by means of applying advanced classification techniques [3, 4]. In view of this, many researchers have applied different signal processing techniques (SPT) in the pre-processing stage along with several classifiers during the classification phase of PQ analysis. The extracted features from the PQE signals during pre-processing analysis can be used to learn and verify the advanced classifiers to get class values of predictions at the final decision phase [3–5].

In the pre-processing stage, several signal analysing methods were used by the researchers for extraction of features from PQEs. Authors [6] applied the spectral analysis of the Fast Fourier Transform (FFT) and Discrete Fourier Transform (DFT) analysis techniques for discriminating of numerous PQEs in power systems. The Short Time Fourier Transform (STFT) in [5] applied for analysis of nonlinear nature of PQEs in power system. These methods are incapable of analysing the nonlinear nature of PQEs [7]. To nullify the issues of these transforms, Wavelet Transform (WT) analysis was widely used by the researchers in PQ study. Because, WT is more flexible in analysing PQEs in both time and frequency domains concurrently [8]. On the other hand, computational burden and being less immune to the noise effect are the biggest issues in the WT approach. In the view of PQEs and fault study applications, the Discrete Wavelet Transform (DWT) of WT series is extensively used [9]. Nevertheless, the choice of mother wavelet for a particular application can be considered as a main challenge in DWT analysis [4]. The researchers extensively used a well-known mother wavelet, namely Daubechies-4 (db4), to analyse PQEs in the majority of the research works [10]. In comparison to the existing SPT, the discrete method of wavelet analysis is broadly utilized since it takes minimum processing time and offers higher accuracy while extracting features in fast manner from the PQE signals [11]. Therefore, in this research work, the application of the DWT technique has been considered for the extraction of features from various PQEs.

Numerous machine learning algorithms were used by the research experts in the classification phase of the PQ studies. The most common techniques include decision trees (DT) [12], Fuzzy logic (FL) based classifiers [13], artificial neural networks (ANN) [14], neural networks with probabilistic function (PNN) [15], Naive Bayes method (NB) [16], K-nearest neighbour (KNN) [14], and SVM [17, 18] have been utilized to classify the PQEs. Among all these classifiers, SVM is one of the most powerful and effective for classifying linear and non-linear data [19]. Additionally, it has superior generalisation performance and is capable of handling an expansive, dimensional input vector proficiently in comparison with other conventional classifiers [5]. Typically, the SVM is useful to avoid over-fitting problem (as encountered in neural networks) and offers the highest accuracy of classification results, especially in high dimensional data sets [20]. Thus, considering the advantages of the SVM classifier, many researchers have applied SVM technique to classify complex PQEs in large power networks as well in MG power systems. Authors [21] used a learning framework which was developed with WT and SVM methods to classify complex PQEs. For the identification of different PQEs in the PV integrated power network, authors [22] applied the SVM learning method with multi-class features. Ray et al. [23] proposed SVM with Independent Component Analysis (ICA) to distinguish between different PQEs in the MG power network. Wang Y et al. [24] applied SVM with Multi Resolution Analysis of DWT to categorise different PQEs. Cortes Robles et al. [25] proposed multi-scale recurrence quantification decomposition (MSRQD) method along with SVM classifier for classification of complex PQEs in grid connected MG system. Furthermore, SVM with different kinds of kernel functions can be used to enhance the classifier performance while solving the non-linear nature of classification problems in PQ study [26]. The kernel function can transform the inseparable data from a small dimensional area to a large dimensional area where the information can be separated more accurately. The different types of kernel functions of SVM include linear kernel, polynomial kernel, and Gaussian kernel (Radial Basis Function), etc. [27]. Biswal et al. [28] proposed a multiclass SVM using linear kernel function with a combination of disturbances versus normal (DVN) approach of feature extraction for classifying complex PQEs in power systems. Radial Basis Function (RBF) and polynomial kernel-based SVM were introduced by the authors of [29] in a hybrid DG environment of a power system network. Similarly, the authors in [30] utilised SVM with RBF based kernel to detect the disturbance patterns in the three-phase simulated signals. Most of the intelligent classifiers, like ANN, PNN, NB, KNN, SVM, and different kernels of SVM, are stated in literature to have their own strengths and weaknesses. For enhancing the precision and generalisation ability of individual weak learners, several ensemble classifiers are used by the researchers. Ensemble classifiers are mainly used to improve the overall performance and stability of weak classifiers through computing their output predictions in different ways [31]. From several research studies, it can be proven that the ensemble approach to classification offers promising results of accuracy compared to individual weak classifiers.

Several ensemble classifiers have been used by researchers to discriminate between different PQEs in conventional and RE integrated power system networks. The Bagging ensemble classifier with the flexible analytic wavelet transform (FAWT) method in [32] is applied to discriminate multiple PQEs in RE connected power networks with promising results compared to individual weak classifiers. The S-Transform extraction method with Adaboost ensemble approach [33] and Hilbert Huang Transform feature extraction with adaptive NFS [34] have been used for PQ analysis with achievement of higher accuracy and better performance than single classifiers. Furthermore, DWT analysis with voting approach in [35] and stacking ensemble approach in [36] have shown better effectiveness in predicting various PQEs in the PV integrated power network. Similarly, to improve the classification accuracy and robustness of individual weak classifiers, the authors in [37] used Random Forest classifier for discrimination of multiple PQ signals in RE connected power network. Thus, it is clear from the literature of the ensemble approach that ensemble models can significantly improve the overall accuracy and generalisation ability of weak classifiers. Hence, in this study, SVM based Random Subspace (RS) ensemble classifier is proposed to discriminate against different PQEs in the MG network. The structure of classifiers used in the RS ensemble method is constructed with different subsets of features which are sampled randomly from the main data set [38]. Because of using randomly selected subset features, the RS method can provide low bias risk with enhancement of prediction performance for the weak classifiers. The RS method also offers superior performance when the training data set has redundant features [39–41].

In most of the research works [2, 4, 23, 24, 42, 43], the PQ analysis in the MG network was carried out either in the on-grid or off-grid (islanded) mode of MG operation. However, to ensure reliable operation and improved PQ of MG network, it is necessary to discriminate between the PQEs in both the on-grid and off-grid modes of MG operation. Also, limited research work was observed with the analysis of PQEs in MG network using an ensemble approach of classification in MG network under the weather intermittence condition of RE sources. Hence, in this study, discrimination of different PQEs is considered in both modes (on-grid and off-grid) of the PV connected MG network under variation of solar PV irradiance with real time condition. From the final results of classification analysis, it is inferred that the proposed SVM based RS ensemble classification model outperforms different types of kernel based SVM classifiers (Linear, Quadratic, and Cubic) in terms of classification accuracy and performance level. The important objectives of this research study are listed below:

- For analysing the causes of different PQEs and evaluating the effectiveness of kernel based SVM learners and the proposed SVM based RS ensemble classifier, a simulation network of the PV connected MG model is formulated with introduction of different PQEs.
- The classification framework is proposed with a combination of DWT technique along with different types of kernel based SVM and RS ensemble classifiers to identify and categorize the various types of PQEs in both the mode of MG network under STC and real time varying solar irradiance of PV conditions.
- The effectiveness of the proposed RS ensemble model has been verified through a comprehensive evaluation of Performance Factors (Kappa Statistics (KS), Recall, Precision, ROC, and F-Measure) results with the kernel based SVM classifiers.

This article is structured as follows: Section 2 explains the definition of MG simulation model with addition of various PQEs, Section 3 describes the concept of the classification framework model, Section 4 presents the detail of data acquisition and signal processing method, Section 5 describes the SVM classifier concept with various kinds of kernel functions (Linear, Quadratic, and Cubic) and the proposed RS ensemble classification model, Section 6 discusses the results analysis based on the classification and performance analysis of the proposed RS ensemble classification model and kernel-based SVM classifiers, Section 7 describes the comparative analysis, and the outcomes with future scope of this study are concluded in section 8.

## 2. Overview of MG model

**Fig 1:**
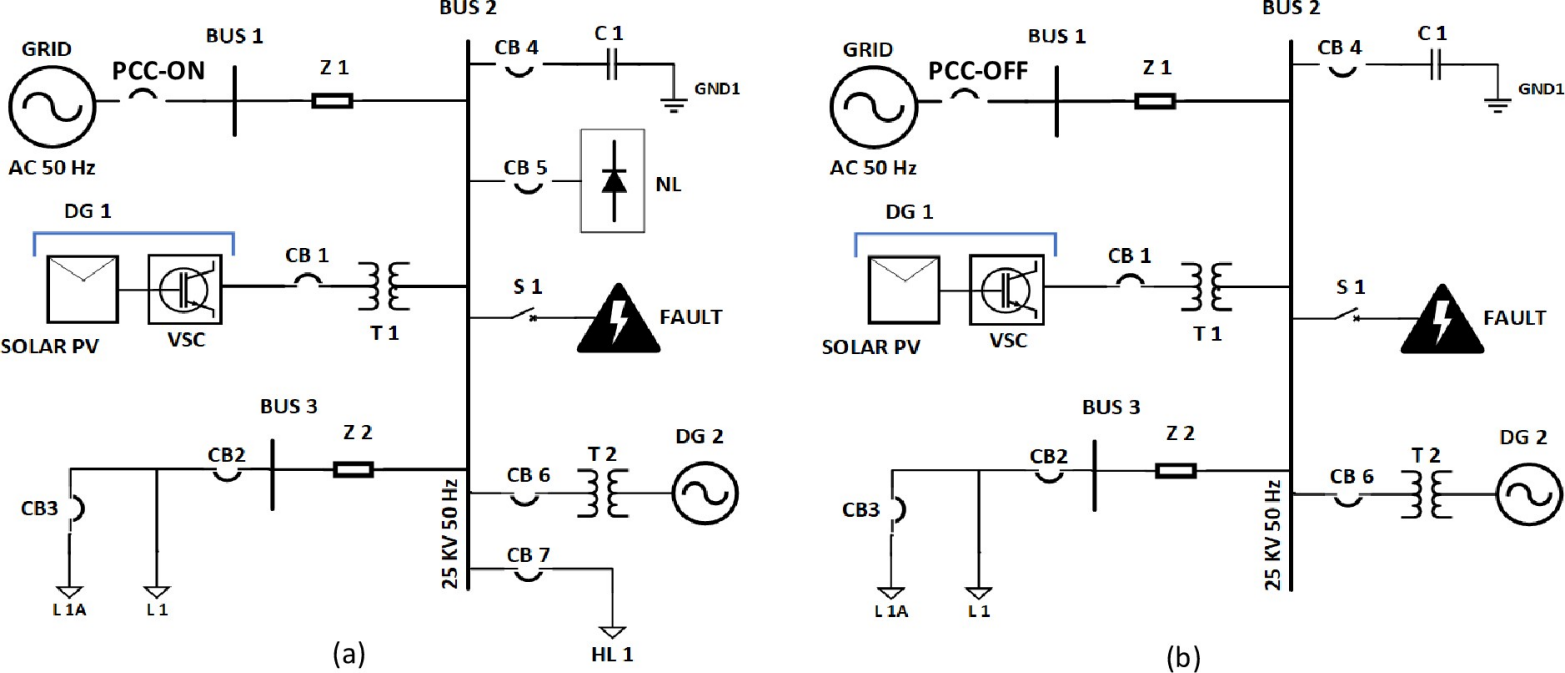
MG Network Model (a) On-grid mode (b) Off grid mode.

The Matlab-Simulink software tool is used to develop a PV integrated MG simulation model. The MG model is simulated with the introduction of different PQE’s (normal, voltage sag & swell, harmonic distortions, and transients (due to switching of capacitor bank, PV inverter, and LG fault) for analysis. The configurations on-grid and off-grid MG models are portrayed in Fig 1A and 1B, respectively. The MG model includes different types of Distribution Generation (DG) sources (Solar Photovoltaic (PV) and diesel powered genset)) and loads (linear and non-linear). Also, the MG model includes with 25 kV feeder lines with a length of 2 km (each). Details of the power ratings of all elements used in the MG network are shown in Table 1.

**Table 1:** Detail of MG components.

**Utility Grid**	Grid Source—G	100 MVA, 25 kV
**PV System**	PV unit—DG-1	250 kWp, 500V dc
	PV Inverter–VSC	250 kVA, 260 V AC
	DG-1 Transformer—T1	300 kVA, 0.26 kV/25 kV
**Diesel Generator**	Synchronous Generator—DG-2	3250 kVA, 2.4 kV
	DG-2 Transformer—T2	2.4 kVA/25 kV, 6000 kVA
**Loads**	Linear Load 1 –L1	2400 kW
	Linear Load 2 (switching)–L1A	500 kW
	Heavy Load (Switching)–HL1	500 kW
	Non-linear Load—NL	Diode Rectifier
**Capacitor**	C1	500 kvar, 25 kV
**Feeders**	Feeder lines 1 & 2 (Z1& Z2) (2 km)	(L_1_) 2.08 mH, (R_1_) 0.0592 Ω,

### 2.1 Description of different PQEs

During PQ analysis, it is considered to follow the threshold limits (as per IEEE 1159 standard [44] of different PQEs in the MG network. Normal and the three most common voltage-related PQEs (sag, swell, and harmonic distortions) are generated by switching heavy (sag/swell) and non-linear (harmonics) loads in the MG network’s off-grid mode. Furthermore, three PQ transients have been generated by switching of capacitor bank (transients-1), PV inverter (transients-2), and ground fault-LG (transients-3) for both modes (on-grid and off-grid) of the MG network. The PQEs with corresponding switching actions are listed in Table 2.

**Table 2:** Switching conditions of different PQEs.

PQEs	Equipment Switching	Duration (s)
Sag	Heavy load–HL1 (CB7 On)	0.4 to 0.6
Swell	Part of Normal load–L1A (CB3 Off)	0.4 to 0.6
Harmonic Distortions	Non-Linear load—NL (CB5 On)	0 to 1
Transients-1	Capacitor bank–C1 (CB4 On)	At 0.4
Transients-2	PV inverter–VSC (CB1 Off)	0.38 to 0.4
Transients-3	Single line to Ground fault—LG (S1 On)	0.4 to 0.43

## 3. Methodology of classification

**Fig 2:**
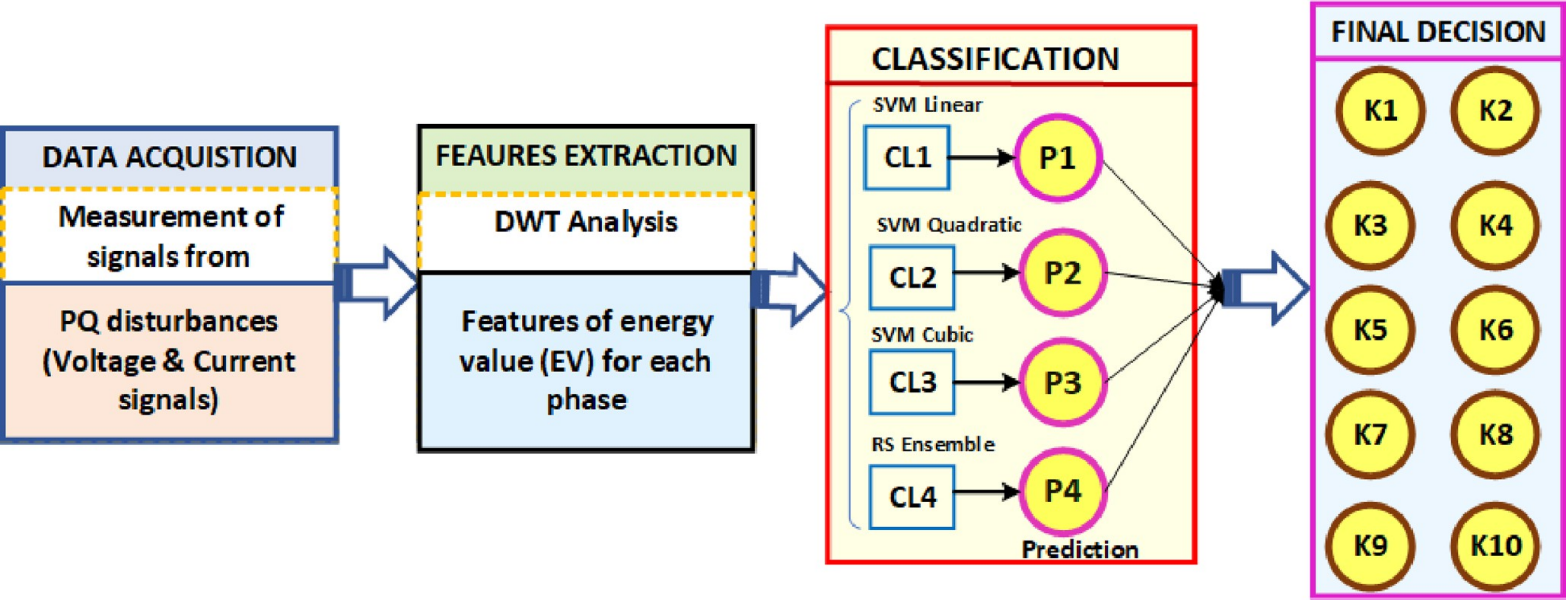
Fundamental concept of proposed classification strategy.

Fig 2 demonstrates the basic methodology for identification and discrimination of various PQEs within the MG network. The main steps in the process of classification are explained below:

**Data Collection (Step-1):** During the simulation of the PV integrated MG model, signal data from different PQEs are collected.

**Feature Extraction (Step-2):** During this step, with the help of the DWT technique, features are extracted from disturbance signal of different PQEs.

**Prediction (Step-3):** To train the kernel based SVM learners and RS ensemble classifier that extracted features are used to get final predictions for further evaluation.

**Final decision (Step-4):** Based on the final predictions, each classifier gives output class labels (K1 to K10) in the final stage.

## 4. DWT method of feature extraction

The Wavelet transform analysis method is one of the most effective methods for decomposing a fast varying signal into numerous sub-components in time and frequency domains [45]. The WT variants are often available in the form of continuous and discrete variants. Continuous wavelet transform (CWT) can be used to address the resolution constraint in STFT, but in the case of real-time applications, it is less beneficial and has low rpetition. The discrete method of wavelet transform (DWT) can be used to nullify the drawbacks of CWT and mathematically can be defined as [46, 47],

$$\text{DWT}\;\left(\text{m},\text{k}\right)=\left[\frac{1}{\sqrt{a_{0}^{m}}}\sum_{n}X\left(n\right)f\left(\frac{k-nb_{0}\;a_{0}^{m}}{a_{0}^{m}}\right)\right]$$ (1)

where $a_{0}^{m}$ is the scaling factor, $nb_{0}\;a_{0}^{m}$ is the translation factor, m and n are the representation of integers, *X(n)* is the time signal, and f is the function of the mother wavelet.

**Fig 3:**
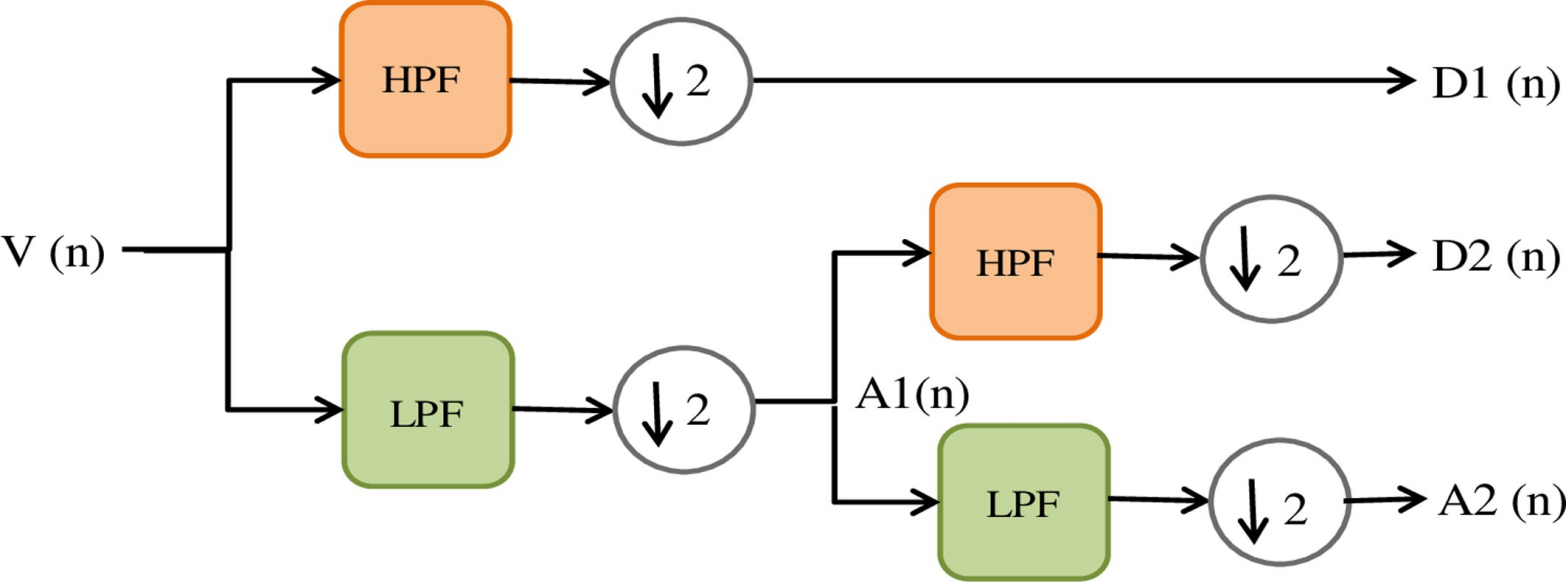
Signal decomposition (stage two).

Multi Resolution Analysis (MRA) is typically used for the DWT process to get wavelet transform coefficients (detail and approximate) through decomposing of the input signal. MRA is more appropriate for decomposing the PQE signals, because it has the characteristics of using less memory and simple implementation. In this process, a series of filter banks are used at each point of decomposition to decompose the signals at different resolutions. Fig 3 indicates the decomposition of the test signal up to the second stage. The input signal V(n) is passed through a collection of high-pass (g1) and low-pass (h1) filters to obtain the detailed (D1, D2) and approximate coefficients (A1, A2). In addition, the signal is downscaled by a factor of two at each step and the approximation of coefficient is used for further decomposition. This decomposition process is carried on till the specified decomposition level is reached [46, 47].

From the below given Eqs (2) and (3), that detail (Di) and approximation (Ai) coefficients can be evaluated:

$$D_{i}\left(k\right)=\sum_{n}g1\left(n\right)A_{i+1}\left(2k+n\right)$$ (2)

$$A_{i}\left(k\right)=\sum_{n}h1\left(n\right)A_{i+1}\;\left(2k+n\right)$$ (3)

where D_i_ and A_i_ are the coefficients of detail and approximation, respectively at the i^th^ level.

where D_i_ and A_i_ denote the detail and approximation coefficients, respectively, at the level of i^th^. High-pass and low-pass filters are associated with the wavelet ω (t) and scaling β (t) functions and can be expressed as,

$$\omega \left(t\right)=\sqrt{2}\sum_{n}g1\left(n\right)\omega \left(2t-n\right)$$ (4)

$$\beta \left(t\right)=\sqrt{2}\sum_{n}h1\left(n\right)\beta \left(2t-n\right)$$ (5)

The mother wavelet of Daubechies-4 (db4) is commonly used in PQ analysis to detect fast transient signals in the power system, according to the literature [48, 49]. Therefore, in this research work, mother wavelet of db4 is considered for analysis of PQE signals.

### 4.1 Evaluation of energy value

The feature extraction is useful in such a way as to provide reduced dimension of the input vector matrix with useful information for the classifiers. Using the Eq (6) [49], the energy value (EV) can be estimated from the detail coefficients of DWT analysis.

$$\text{Energy}\;\text{Value}\;\left(\text{EV}\right)=\frac{1}{K}\sum_{i=1}^{k}{\left\lceil D_{ji}\right\rceil }^{2}$$ (6)

let mean $\mu_{i=}\frac{1}{K}\sum_{i=1}^{K}D_{ji}$, j = 1,2,3….l (decomposition level), K is the number of samples for each decomposed signal.

## 5. Materials and classification methods

In this research, the software tool WEKA is utilised to discriminate between different PQEs in the MG network using extracted features. The WEKA is an effective tool with inclusion of several classification algorithms and the option of providing base and ensemble classification, clustering, and visualization facilities [10]. In this study, different types of kernel based SVM classifiers such as SVM linear kernel, SVM polynomial (quadratic and cubic), and SVM based RS ensemble classification approach have been considered to classify various PQEs like normal, sag, swell, and distortion of harmonics with consideration of class labels such as K1, K2, K3, and K4, respectively, in off-grid mode of the MG network. In addition, other PQEs like three numbers of PQ transients (due to switching of capacitor bank and PV inverter, and LG fault) have been classified in both modes (off-grid and on grid) of the MG network with consideration of the following class labels: K5, (transient 1) K6 (transient 2), K7 (transient 3) in off-grid and K8 (transient 1), K9 (transient 2), K10 (transient 3) in on grid, respectively.

The estimated energy values from the extracted features of various PQE signals have been utilised to learn the kernel based SVM learners during the first phase of classification. While learning the classifiers, a k-fold cross validation method is applied with the input data set to nullify the issue of over-fitting. The prediction capability of classifiers can be assessed with the help of the cross validation method [50]. The training data (X) is separated into equal sized chunks with a bunch of k disjoint subsets (X1, X2, …., Xk). From available k-subsets, one subset is utilised for testing, and the remaining subsets (k-1/k) are utilised for classifier training [50]. In this work, cross validation with 10 folds is considered while learning the classifiers. This section describes the kernel based SVM classifiers (linear, polynomial (quadratic and cubic)), and the proposed RS ensemble classifier in more detail.

### 5.1 SVM classifier

**Fig 4:**
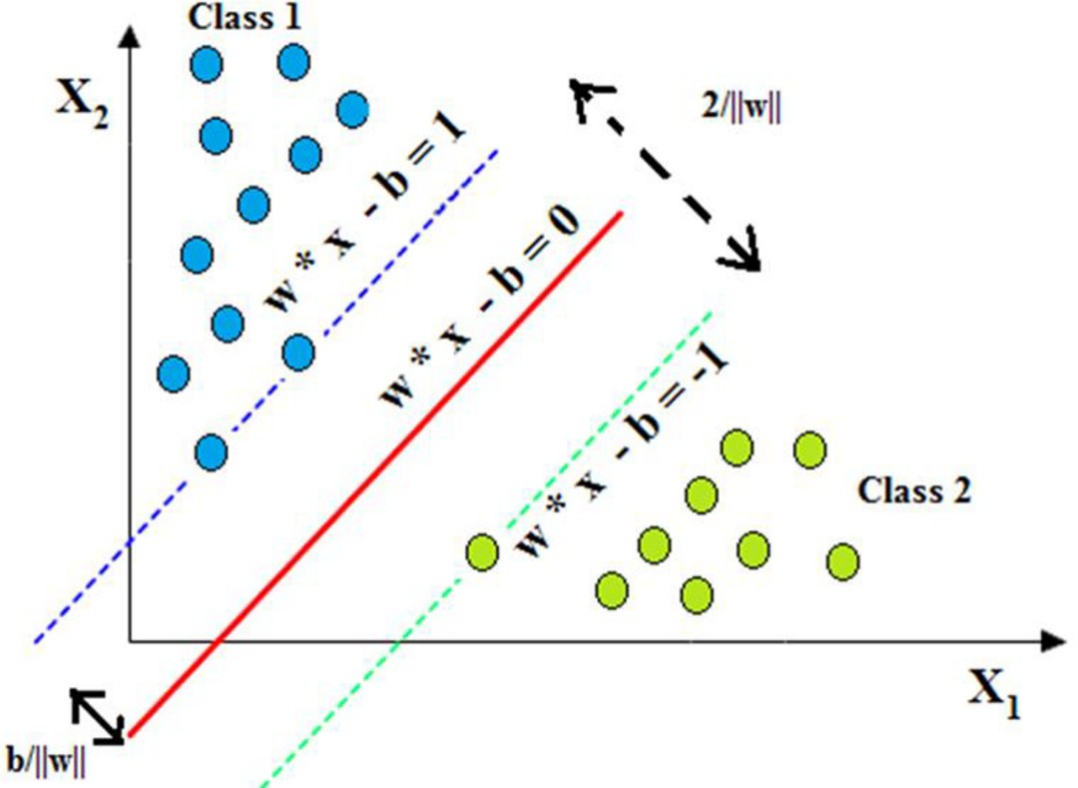
Concept of SVM classifier.

SVM is a more flexible machine learning algorithm for the applications of pattern recognition and classification [4]. The SVM rule algorithm was developed by Vapnik [51] and operates on the basis of supervised learning theory. SVM seeks to separate the heperplane in an optimum way by maximising the margin data set and hyperplanes [52]. It offers good generalisation accuracy on unknown data and supports the intensive optimization methods that enable SVM to learn from a large scale of data [53]. An example of the SVM concept is shown in Fig 4.

For a given training data set, $\{x_{i},\;y_{i}{\}}_{i-1}^{K}$, where *x*_*i*_ ∈ *R*^*n*^ is the vectors of input data, *y*_*i*_ ∈ {+1, -1} denotes different classes, and K is the number of samples. The given training data set can be separated linearly by the hyperplane f(x), as represented by Eq (7) [52–54]

$$\text{f}\left(\text{x}\right)=w.x+b=\;\sum_{N=1}^{K}\left(w_{N}.x_{N}\;+b\right)$$ (7)

where w and b represent the terms for weights and bias used to optimise the position of the hyperplane separation. The constraints as given in Eq (8) should be satisfied to separate the hyperplane.

$$y_{i}f\left(x_{i}\right)=y_{i}\left(w.x_{i}+b\right)\geq 1,\;\left(i=1,2,3,\ldots .,\;K\right)$$ (8)

It is possible to estimate the distance between margin and vectors *x*_*i*_ that lies on the incorrect side of the margin is generally outlined by the positive slack variable £_*i*_. For separating given data, optimal hyperplane is determined by solving the optimization problem which is expressed in Eq (9):

$$\text{To}\;\text{Minimise},\;\frac{1}{2}{\left\|w\right\|}^{2}+\text{C}\;\sum_{i=1}^{K}{£}_{i},\;i=1,2,3,\ldots .,\;K$$ (9)

Prone to *y*_*i*_(*w*.*x*_*i*_ + *b*) ≥ 1 - *£*_*i*_, *and* *£*_*i*_ ≥ 0, let C denote the penalty for error, and by using Lagrangian multipliers *α*_*i*_, the problem of optimization (Eq (9)) will be transformed into a problem of dual quadratic optimization, as expressed in Eq (10) [54]:

$$\text{To}\;\text{Maximise},\;\text{L}(\alpha )=\sum_{i-1}^{K}\alpha_{i}-\frac{1}{2}\sum_{j=1}^{K}\alpha_{i}\alpha_{j}y_{i}y_{j}\left(x_{i},\;y_{i}\right)$$ (10)

Subject to $\sum_{i-1}^{K}\alpha_{i}y_{i}=0,\;and\;\alpha_{i}\geq 0$, The problem of dual optimization is possible to solve by using linear decision function, expressed in the Eq (11):

$$\text{f}\left(\text{x}\right)=\text{Sign}\left(\sum_{ij=1}^{K}\alpha_{i}y_{i}\left(x_{i},\;x_{j}\right)+b\right)$$ (11)

**Fig 5:**
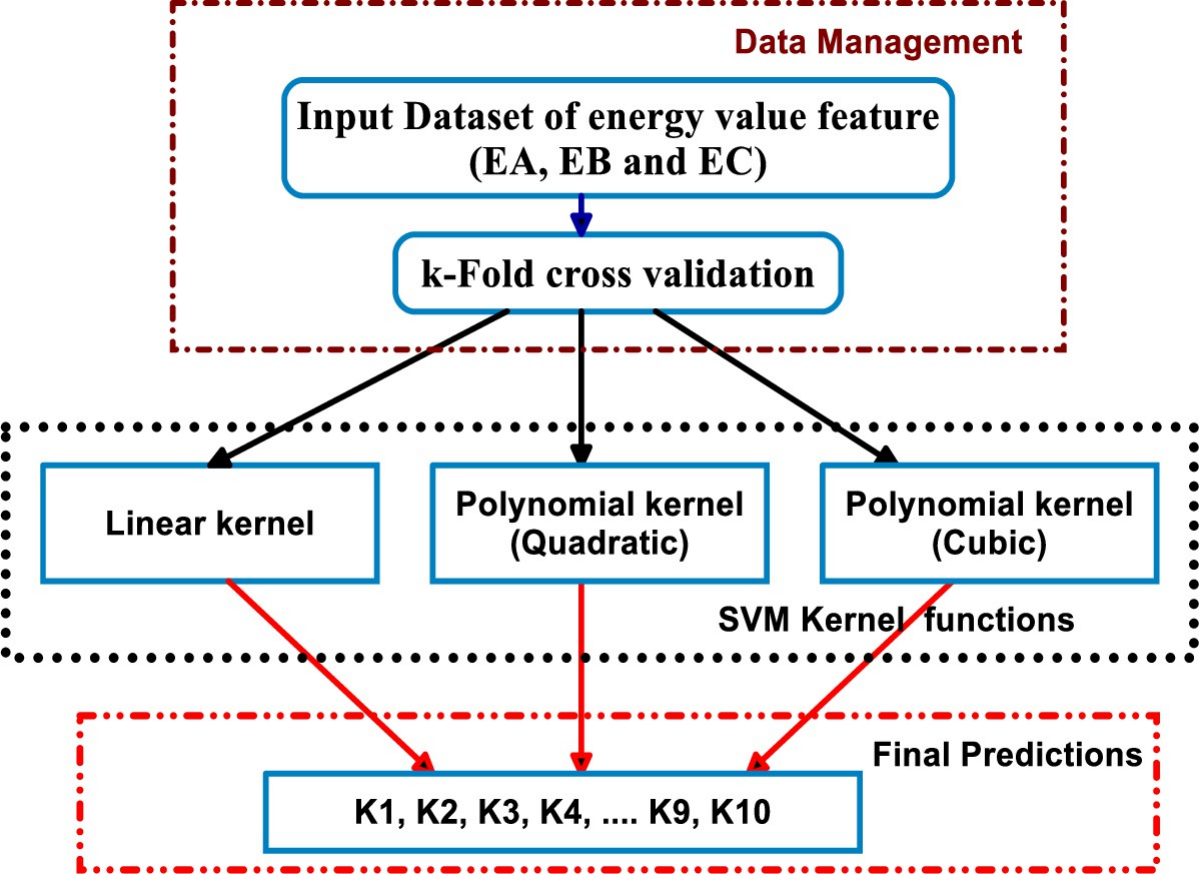
Classification framework of kernel based SVM classifiers.

The kernel functions of SVM are useful for solving nonlinear classification problems. By using a nonlinear function (*φ*), the kernel functions of SVM can be used to transform inseparable data from low-dimensional space to a higher-dimensional space where the data is separated linearly [52]. The function of non-linear decision with kernel (K) inclusive can be defined as follows:

$$\text{f}\left(\text{x}\right)=\text{Sign}\left(\sum_{ij=1}^{M}\alpha_{i}y_{i}k\left(x_{i},\;x_{j}\right)+b\right)$$ (12)

where *k*(*x*_*i*_, *x*_*j*_) is the kernel function that can be written as ɸ(*x*_*i*_) and ɸ(*x*_*j*_), respectively. In this study, SVM classifiers with different kernel functions like linear, olynomial (quadratic and cubic), and RBF (Gaussian fine) have been used to categorise various PQEs in the MG model of power network. Furthermore, for classification of multi class PQEs in MG network that kernel based SVM classifiers have been used with adoption of the One Against One (OAO) multiclass method [55]. The classification of various PQEs in the MG network using kernel based SVM classifiers is shown in Fig 5. A 10 folds cross validation method is applied with a given input data set (400 instances (40 instances per PQE) and three features) while learning kernel based SVM classifiers (linear kernel, and polynomial kernel (Quadratic & Cubic)). In the final decision phase, predictions of class values are obtained from each classifier.

#### 5.1.1 SVM linear kernel

The linear kernel is a simple and easy to interpret kernel function. It is a fast data mining algorithm for solving multiclass classification problems. It can be used for a number of features in a large data set. The linear kernel function can be expressed as [56],

$$k\left(x_{i},\;x_{j}\right)=x_{i}{}^{T}x_{j+C}$$ (13)

where *k*(*x*_*i*_, *x*_*j*_) is the kernel function, *x*_*i*_ and *x*_*j*_ are feature space vectors. and ‘C’ is the box constraint or regularization parameter. The value of regularization parameter (C) is greatly influences over the trade off between the maximisation of classification margin and minimisation of error [57]. In this study, for the linear kernel of SVM, the value of ‘C’ is considered as 9 on the basis of achieved higher accuracy and minimum error level. The steps of classification process with linear kernel of SVM classifier are illustrated in Table 3.

**Table 3:** Process steps of classification: SVM linear kernel.

Input: Training Data (D) = Input energy values; response class names Input features (X_i_) = {’Energy-A’, ’Energy-B’, ’Energy-C’}; Response class (Y_i_) = {’K1’,’K2’,‥,’KN’ }; N = 10 Output: Predictions and accuracy (%) from SVM linear kernel Perform cross-validation (10-fold) **Step 1:** Randomly divide data set X into k subsets of equal size: X = (X1, X2, X3, ‥, XK); (K = 10); **Step 2:** For k← 1 to 10; Train the classifier SVM linear kernel, from D or DK **Step 3:** Apply Kernel Function’, ’linear’, (Eq (13)) Set ’Polynomial Order’, {0}, Assign ’Box Constraint’, for Linear {9}, ’Standardize’, true, Multi-class approach’, One Against One (OAO); End for Create the result structure with predict function **Step 4:** Validate predictions = class names (‘K1’, ‘K2’, …,’K10’); **Step 5:** verify accuracy = Classification Accuracy (%); End for

#### 5.1.2 SVM polynomial kernel

It is a global kernel with good generalization ability. Ii is useful for learning high dimensional data with nonlinear boundaries, and its kernel parameters have a substantial effect on the decision boundary. This kernel is capable of solving multi class problems with allowable margin [58]. The definition of a polynomial kernel can be expressed as [56],

$$k\left(x_{i},x_{j}\right)={\left(x_{i}{}^{T}x_{j}+C\right)}^{d}$$ (14)

where ‘C’ is the regularisation or box constraint parameter; *k*(*x*_*i*_, *x*_*j*_) is the kernel function; *x*_*i*_ and *x*_*j*_ denotes feature space vectors; and ‘d’ states the degree of polynomial function. The Quadratic and Cubic kernels are the sub types of polynomial kernel functions of SVM. The quadratic kernel is a 2^nd^ order polynomial kernel function that can be stated as [59],

$$k\left(x_{i},\;x_{j}\right)={\left(x_{i}{}^{T}x_{j}+C\right)}^{2}$$ (15)

The cubic kernel is a third order polynomial kernel function and it can be defined as [59, 60],

$$k\left(x_{i},\;x_{j}\right)={\left(x_{i}{}^{T}x_{j}+C\right)}^{3}$$ (16)

For polynomial kernel based SVM classifiers (quadratic and cubic), two parameters like regularisation parameters ‘C’ and ‘d’ degree of polynomial function are greatly influenced by their performance level [57]. In this work, according to the observation of higher accuracy and minimum level of mean absolute error of classification, the value of ‘C’ is considered as 12 for both quadratic and cubic kernel based SVM classifiers, and the value of ‘d’ is considered as 2 for quadratic and 3 for cubic kernel based SVM classifiers, respectively. The steps of classification process with the polynomial kernel of SVM classifier are illustrated in Table 4.

**Table 4:** Process steps of classification: SVM polynomial kernel.

Input: Training Data (D) = Input energy values; response class Names Input features (X_i_) = {’Energy-A’, ’Energy-B’, ’Energy-C’}; Response class (Y_i_) = {’K1’,’K2’,‥,’KN’ }; N = 10 Output: Predictions and accuracy (%) from SVM linear kernel Perform cross-validation (10-fold) **Step 1:** Random splitting of data set X into k times with equal size of subsets: X = (X1, X2, X3, ‥, XK); (K = 10); **Step 2:** For k← 1 to 10; Train the classifier SVM polynomial kernel, from D or DK **Step 3:** Apply Kernel Function’, ’Quadratic’, (Eq (15)) / ‘Cubic’, (Eq (16)), Set ’Polynomial Order’, for Quadratic {2.0} / for Cubic {3.0}, Assign ’Box Constraint’, for Quadratic {18} / for Cubic {18}, ’Standardize’, for Quadratic true / for Cubic true, Set ‘Multi-class approach’, One Against One (OAO); End for Create the result structure with predict function **Step 4:** Validate predictions = class names (‘K1’, ‘K2’, …,’K10’); **Step 5:** validate accuracy = Classification Accuracy (%); End for

### 5.2 Random subspace (RS) ensemble classifier

**Fig 6:**
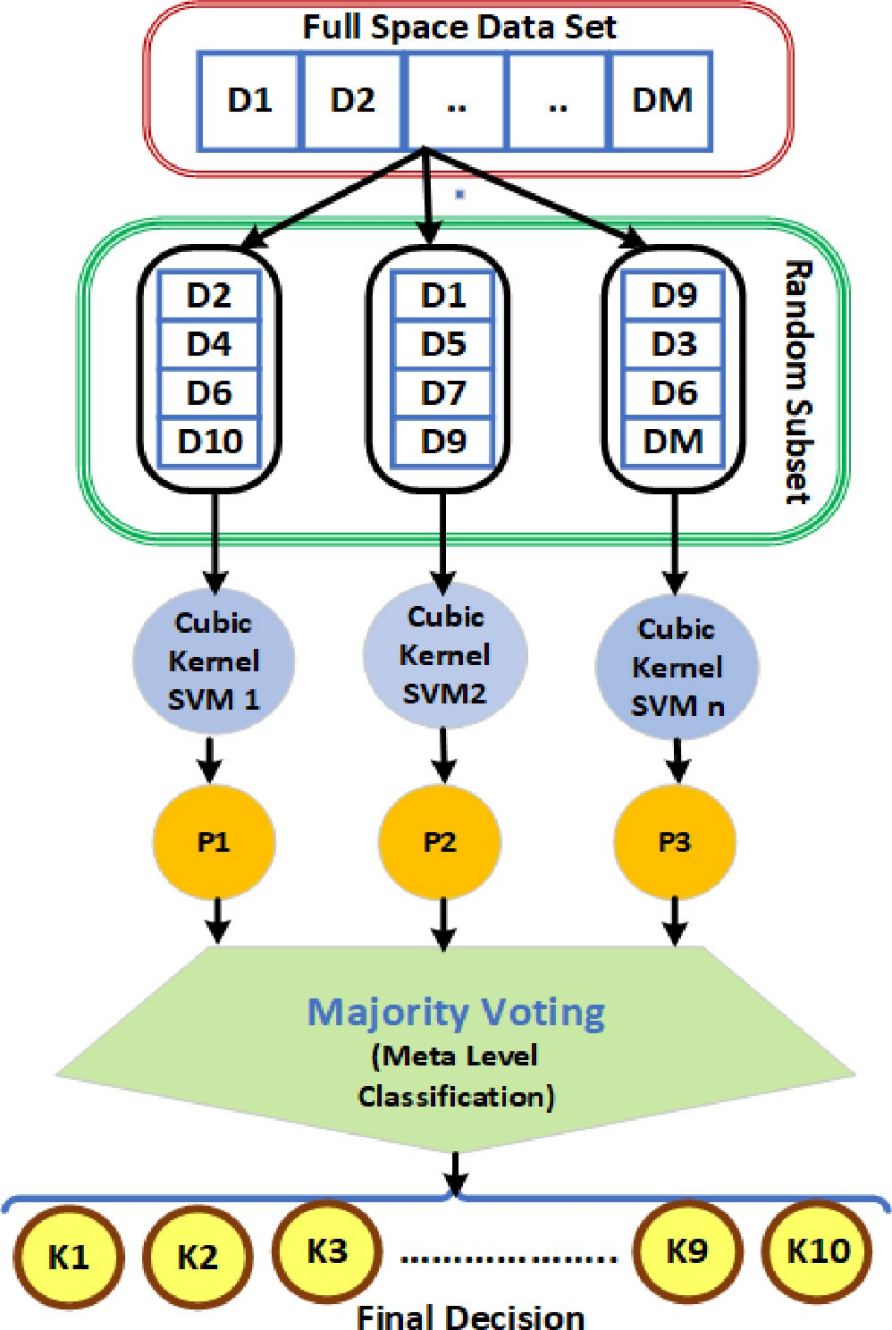
Basic configuration of RS ensemble model.

The RS ensemble classifier can achieve the benefits by applying a random subset of features over the combined set of base classifiers. Fig 6 depicts the basic configuration of RS ensemble model. Randomly selected subset features (between D_1_ and D_M_) from the complete space data set (D) are utilised to learn the set of N number of base classifiers in the model of the RS ensemble approach [53]. A majority voting rule is implemented over the output predictions of weak classifiers to obtain target class labels at final stage of classification [38]. The performance and accuracy precision of weak classifiers are improved by the ensemble approach of RS technique to effectively exploit their outcome predictions. Furthermore, because the classifiers are easily trained using smaller subspaces with the RS technique, the features to instance ratio can be significantly improved [38].

For this RS ensemble model, the p* dimension feature subset (p*<p) is randomly chosen from a given p-dimensional data set for PQ analysis. Following that, the suitable weak classifier is learned using the subspace feature vectors. This approach is repeated M times in order to train M classifiers with a new subset of feature vectors each time. Finally, majority voting is used to evaluate the predictions of N classifiers. The RS method process steps are explained in further level in Table 5 [39, 40].

**Table 5:** Process steps of classification: RS ensemble classifier.

**Input: Training data set,** $\text{D}=\left\{{\text{D}}_{1}{,\;\text{D}}_{\text{2}}{,\;\ldots ,\text{D}}_{\text{m}}\right\};\;\left(\text{Let}\;\text{D}=\text{X}\right)$ (17), **Step 1:** Consider each set of training sample K_j_ has a p-dimensional feature vector, written as, ${\text{K}}_{\text{j}}\text{=}\left\{{\text{K}}_{\text{j1}}{,\;\text{K}}_{\text{j2}}{,\ldots ,\text{K}}_{\text{jp}}\right\};\left\{\text{j}=\left(1,2,\ldots ,\text{m}\right)\right\}$ (18), **Step 2:** Randomly selects feature elements (p*< p) from p-dimensional feature vector T_j_, Then, (i) Training sample of original set X becomes X^r^, and denoted as, X^r^ = {K_1_^r^, K_2_^r^, …,K_m_^r^} (19), (ii) Each training sample in X^r^ consists of p*-dimensional feature elements, and stated as, X^r^ = {K_1_^r^, K_2_^r^, …,K_p*_^r^} (20), **Step 3:** Select the feature element X_jk_^r^ {T = (1,2,…, p*)}in random with uniform distribution, **Step 4:** Build M classifiers in RS with X^r^, as C^m^(x), {m = (1,2,…,M)}; M = 10, **Step 5:** Apply majority voting rule over the output predictions of classifiers, and voting rule is stated as, $\text{h}\left(\text{x}\right)=\text{arg}\;\text{max}\;\sum_{j=1}^{n}C^{m}\left(x\right),\;y$ (21), y∈[1,−1] where y ∈ [1, –1] states the decision of class label, C^m^ is the ensemble size of classifier. End for Create the result with final output predictions **Step 6:** Validation of predictions = class labels (‘K1’, ‘K2’, …,’K10’); **Step 7:** Evaluate accuracy = Classification Accuracy (%); End for

In general, the output classification performance of the RS ensemble technique is determined by the two main factors, such as size of the feature subset (subspace) and the number of weak classifiers (ensemble size). For this research work, SVM based (cubic kernel) RS ensemble classification model is proposed to discriminate different PQEs in PV connected MG network with both modes of operation (on-grid and off-grid mode) of MG network. To get better performance, the sub space size of 0.5 and 10 number of weak classifiers (SVM cubic kernel) are assigned for the proposed RS ensemble model.

### 5.3 Description of performance factors

The definitions of important PF which are used to evaluate the effectiveness of classifiers are given below:

- **Kappa Statistics (KS):** KS can be used to estimate the consistency between the actual and targeted PQEs. According to the estimated KS value, the range of performance can be decided as excellent (KS value 1), good (KS value 0.4 to 0.75), and poor (KS < 0.4), respectively. The expression of KS term is defined as [36],
$$\text{KS}=\frac{\text{Actual}\;\text{PQEs}-\text{Targeted}\;\text{PQEs}}{1-\text{Targeted}\;\text{PQEs}}$$ (22)
- **Precision (P):** It is a ratio between of correctly predicted observations (true positives) and the sum of total predicted observations (true positives + false positives), and it can be expressed as below [36]:
$$\text{P}=\frac{T_{P}}{T_{P}+F_{P}}$$ (23)
  where *T*_*P*_ denotes the true positive and *F*_*P*_ denotes the false positive
- **Recall (R):** It is a ratio between correctly predicted observations (true positives) and sum of all observations including true positives and false negatives and it can be defined as [36],
$$\text{R}=\frac{T_{P}}{T_{P}+F_{N}}$$ (24)
- **F-measure:** The weighted average of precision and recall is called as F-measure and the expression of F-measure can be defined as [36],
$$\text{F-measure}=\frac{\left(2*\left(Precision*Recall\right)\right)}{Precision+Recall}$$ (25)
- **Receiver Operating Characteristic (ROC):** The performance of a classifier can be determined on the basis of the area under the curve of ROC. If the area under the curve reaches 1 or nearer, then the performance of classifier is the best and most accurate [36].

## 6. Results and discussion of PQ analysis

**Fig 7:**
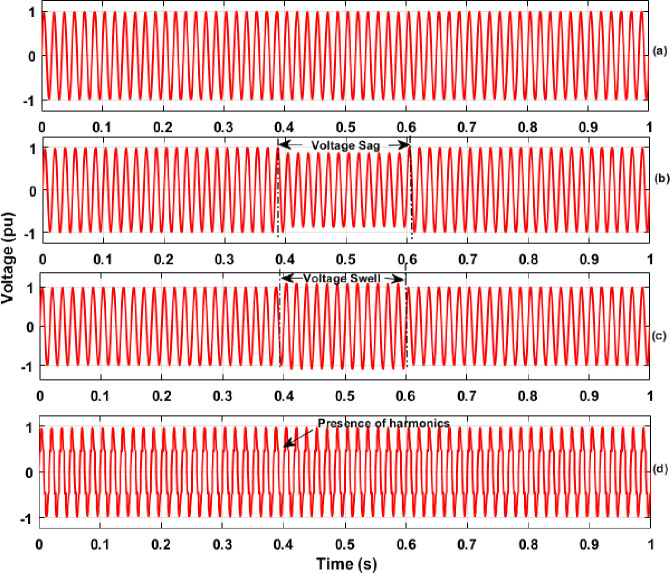
Three phase voltage signals in off-grid mode of MG network (a) Normal; (b) Sag; (c) Swell; (d) Distortion of Harmonics.

**Fig 8:**
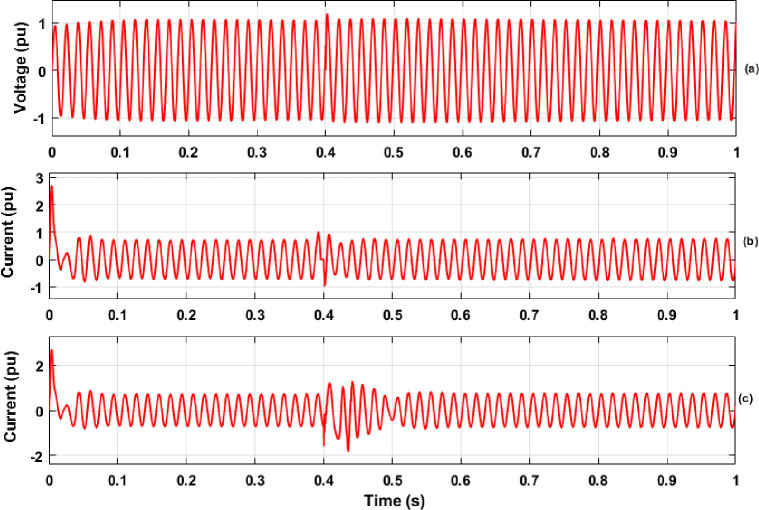
Voltage and Current signals in off-grid mode of MG network (a) Voltage Transients-1 (switching of capacitor); (b) Current Transients-2 (switching of PV Inverter); (c) Current Transients-3 (LG Fault).

**Fig 9:**
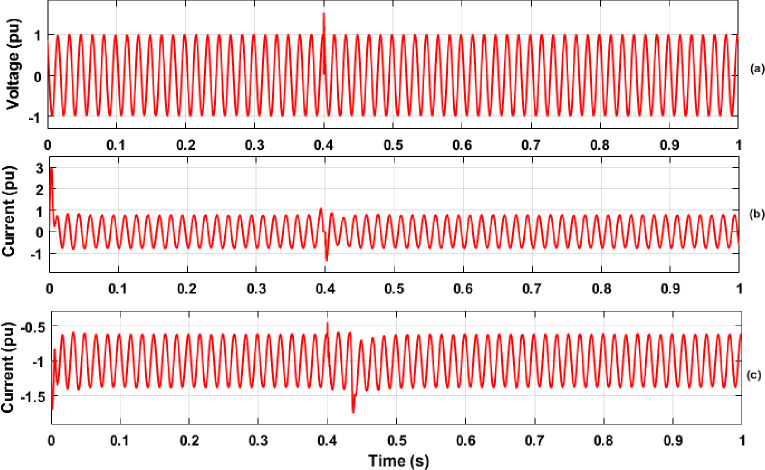
Voltage and Current signals in on-grid mode of MG network (a) Voltage Transients-1 (Switching of capacitor); (b) Current Transients-2 (Switching of PV Inverter); (c) Current ransients-3 (LG Fault).

In this section, the results of PQ analysis that includes extraction of features with the DWT method and classification of various PQEs in the MG network are discussed in detail. The simulation was carried out for the proposed MG model with creation of various PQEs in both the off-grid and on-grid modes of MG network under STC and variation of solar PV irradiance with real time conditions. During simulation analysis, the simulation time of 1 sec was considered for each case of PQEs studied. The switch conditions for the creation of various PQEs along with details of time span for the occurrence of each PQE in both modes of MG network are illustrated in Table 2 of Section 2.1. From the disturbance signals (three phase voltage and current) of various PQEs, features of energy values were extracted with the help of DWT method. As an example, the single phase (phase-A) voltage and current signals during the case of each PQE (in the grid connected and islanded MG network) are shown in Figs 7–9, respectively. The results of DWT analysis and the results of the proposed RS ensemble and kernel based SVM classifiers are discussed in detail as follows.

### 6.1 Results and discussion of DWT analysis

**Fig 10:**
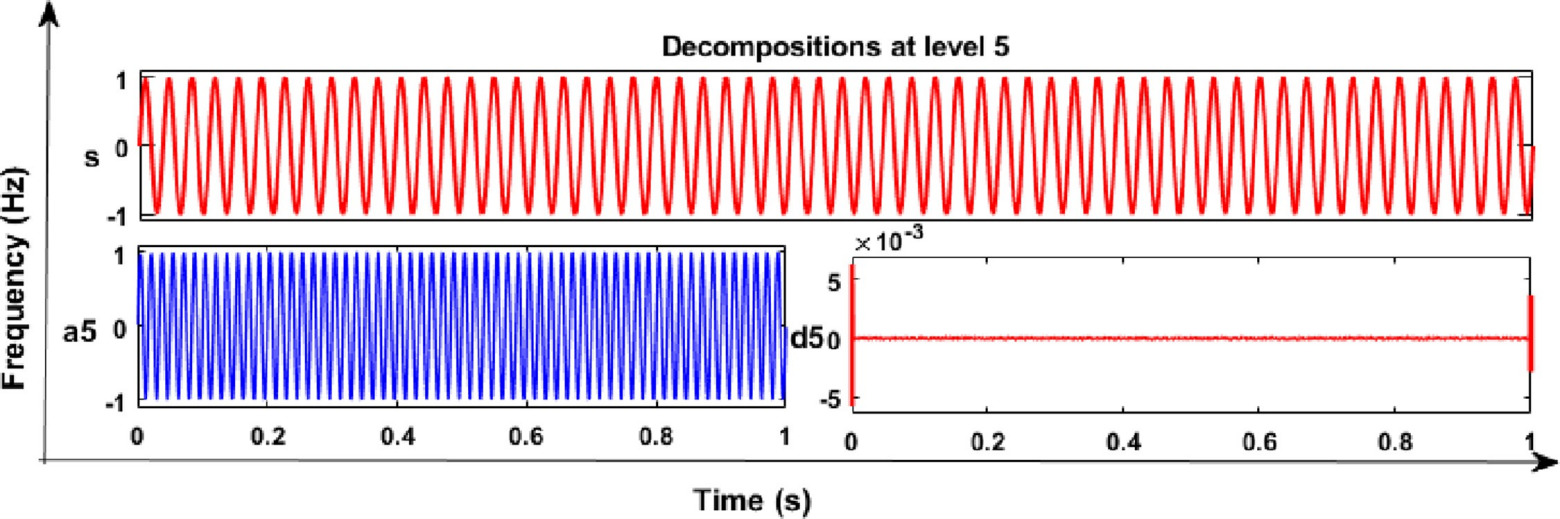
DWT analysis in off-grid MG: Normal voltage signal.

**Fig 11:**
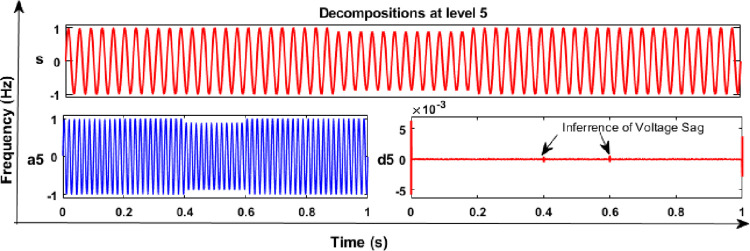
DWT analysis in off-grid MG: Voltage sag signal.

**Fig 12:**
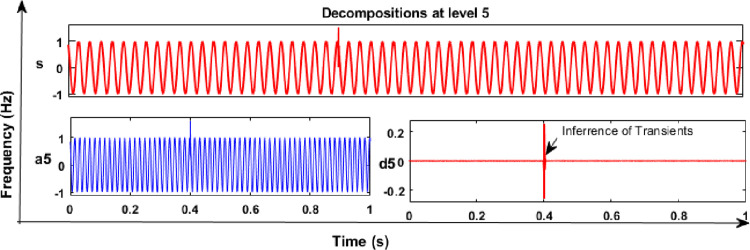
DWT analysis in on-grid MG: Voltage transients-1 signal.

From the DWT analysis, extracted features of energy values of various PQEs in the MG network were used to learn the proposed RS ensemble and kernel based SVM classifiers like linear, polynomial (quadratic, and cubic). The main factors such as mother wavelet (db4), decomposition level (5^th^), and sampling frequency (24 Hz) were considered during the analysis of PQE signals with the DWT method. From the analysis of all PQE signals, wavelet coefficients of detail (d1 to d5) and approximation (a5) were obtained for further analysis. As an example, captured 5^th^ level decomposition of voltage signals of PQEs (normal and sag in phase A) in the off-grid mode of MG network are shown in Figs 10 and 11, respectively. Similarly, in the on-grid mode of MG network, that captured decomposition of voltage transients 1 signal (switching of capacitor) in phase C is shown in Fig 12.

From the results of DWT analysis, it can be concluded that the value of wavelet transform coefficient (d5) has a small magnitude for normal signal and its level of magnitude varies abruptly during the period of voltage sag and transient conditions. Similarly, the decomposition analysis was also carried out for other PQEs and transient signals in all three phases of the MG network. Finally, the extracted coefficients from the voltage and current signal of different PQEs were utilised to evaluate the energy values, using Eq (6).

### 6.2 Results of classification analysis

The extracted energy values of the input data set (400 samples) with adoption of 10-fold validation method were applied to learn the kernel based SVM learners (linear kernel, polynomial kernels (quadratic and cubic)) and SVM based RS ensemble classifier. In this study, the common PQEs (normal, sag, swell, and distortion of harmonics) in off-grid mode of MG network and PQ transients (Transient 1, transient 2, and transients 3) in both off-grid and on-grid mode of MG network were classified by kernel based SVM learners and RS ensemble model under STC and variation of solar irradiance of PV with real time condition. During this analysis, around 400 numbers of instances (40 instances per PQE) were considered to learn the classifiers. In general, the classification accuracy (CA) of classifiers as given in Eq (26) is defined as the ratio between correctly predicted PQEs and the total number of PQEs studied. From the classification analysis, the effectiveness of the proposed RS ensemble classifier is verified (in terms of CA) with the results of individual kernel based SVM classifiers.


$$\text{CA}=\frac{\text{Correctly}\;\text{predicted}\;\text{PQEs}}{\text{Total}\;\text{PQEs}}\times 100\%$$ (26)

#### 6.2.1 Results of classification with kernel based SVM classifiers

**Fig 13:**
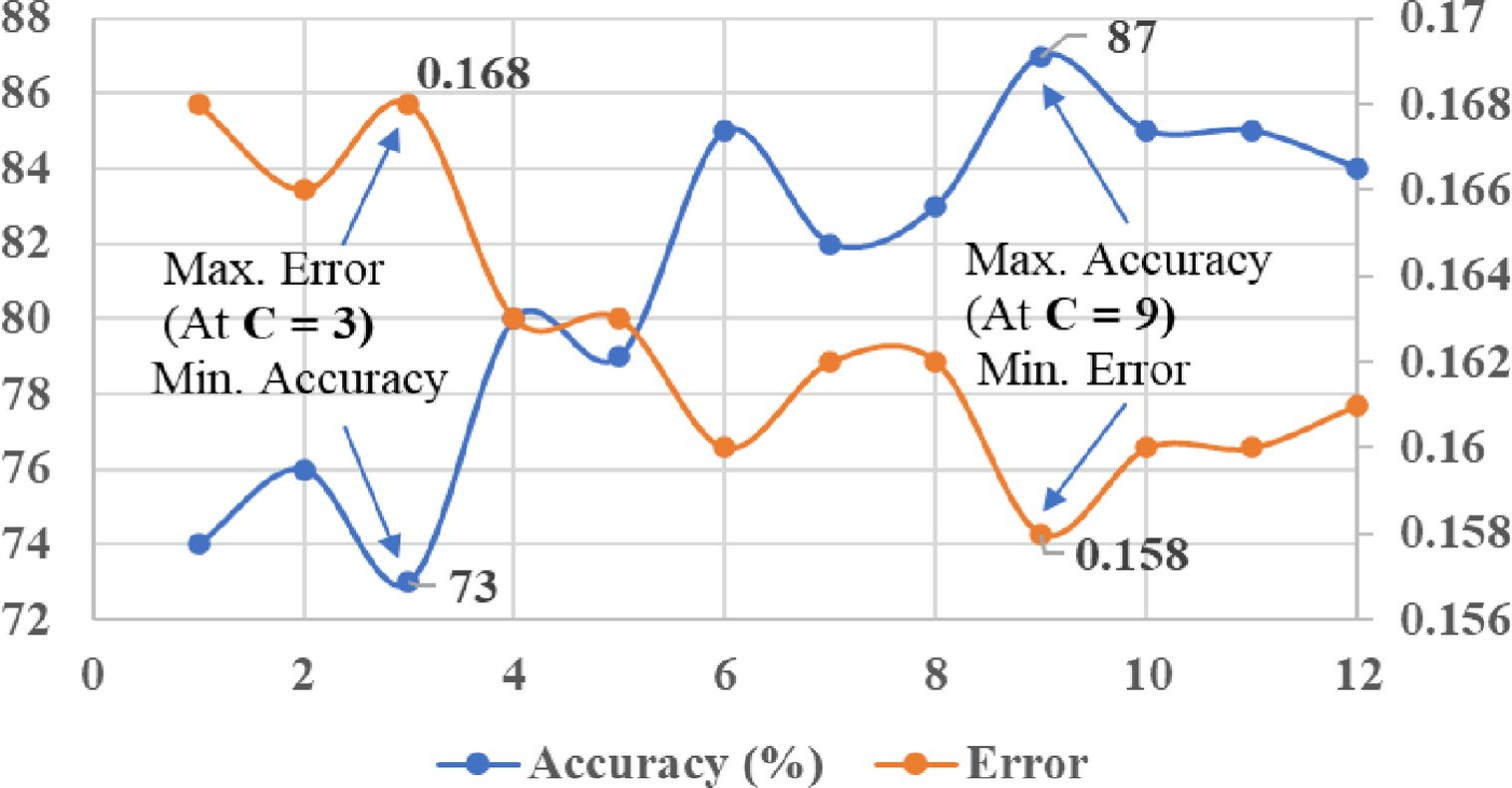
Tuning of kernel parameter (C) with linear kernel of SVM.

**Fig 14:**
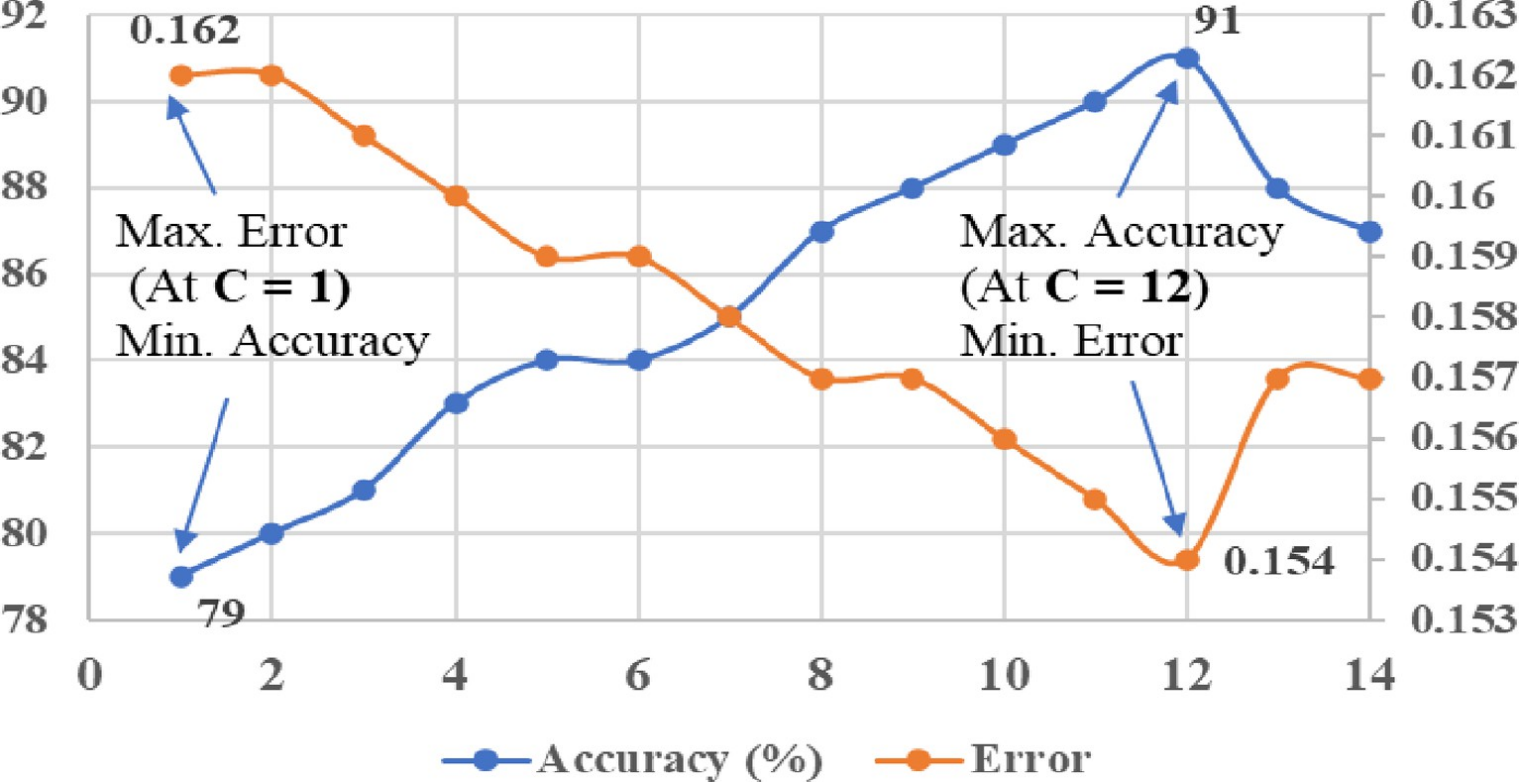
Tuning of kernel parameter (C) with quadratic kernel of SVM.

**Fig 15:**
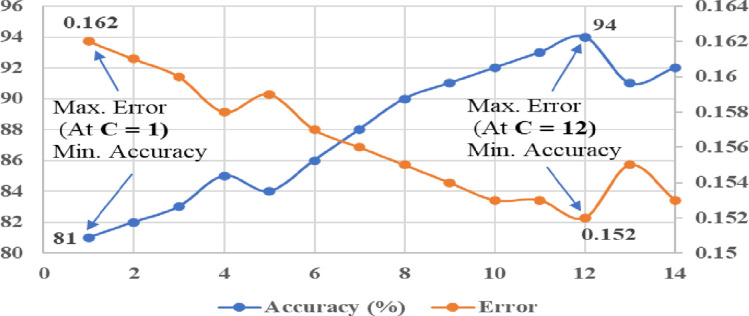
Tuning of kernel parameter (C) with cubic kernel of SVM.

In order to achieve effective performance of kernel based SVM classifiers, it is very important to select the appropriate value of penalty or regularisation parameter “C” while classification. Changing of value “C” can influence the performance accuracy, classification error, and margin of hyperplane in SVM. In this study, the penalty factor “C” was tuned manually on the account of getting minimum classification error and maximum accuracy of kernel classifiers. For the linear kernel SVM classifier, the tuning range of “C” value was considered between 1 and 12 (with steps of 1). The tuning of “C” value for the linear SVM classifier with respect to the classification error and accuracy is shown in Fig 13. From the results of tuning (Fig 13), it can be noticed that maximum classification accuracy (87%) and minimum error (0.158) were achieved at the “C” value of 9. Similarly, the tuning range of “C” value was considered between 1 to 14 (with steps of 1) for the polynomial kernels (Quadratic and Cubic) of SVM classifiers. From the tuning results of “C”, as shown in Figs 14 and 15, maximum accuracy (91% and 94%) and minimum error (0.154 and 0.152) were achieved at the “C” value of 12 with Quadratic SVM and Cubic SVM classifiers, respectively.

*(a) SVM classifiers*: *Classification results under STC of solar PV*. In this case, the PQEs in both the on-grid and 0ff-grid mode of the MG network were classified with kernel based SVM classifiers under STC (PV cell temperature of 25°C, irradiance (1000 watts/m^2^), and air mass of 1.5 M). For analysis, around 400 numbers of instances of all PQEs (40 instances per PQE) were considered for analysis. The results of the confusion matrix for all the kernel based SVM classifiers (Linear, Quadratic, and Cubic) are given in Table 6. The correctly and incorrectly classified instances of each PQE in the confusion matrix are represented as diagonal and off diagonal elements, respectively. Furthermore, the details of all the classified instances for all the PQEs in the MG network are given in Table 7.

**Table 6:** Results of confusion matrix: Kernel based SVM classifiers.

SVM Linear Kernel											
Class	K1	K2	K3	K4	K5	K6	K7	K8	K9	K10	MG Mode
K1	√	0	0	0	0	0	0	0	0	0	
K2	0	√	X	0	0	0	0	0	0	0	
K3	0	X	√	0	0	0	0	0	0	0	
K4	0	0	0	√	0	0	0	0	0	0	Off-Grid
K5	0	X	X	0	√	0	0	0	0	0	
K6	0	X	0	0	0	√	0	0	0	0	
K7	0	0	0	0	0	0	√	0	0	0	
K8	0	0	0	0	0	0	0	√	X	0	
K9	0	0	0	0	0	0	0	0	√	0	On Grid
K10	0	0	0	0	0	0	0	0	0	√	
SVM Quadratic Kernel											
Class	K1	K2	K3	K4	K5	K6	K7	K8	K9	K10	MG Mode
K1	√	0	0	0	0	0	0	0	0	0	
K2	0	√	0	0	0	0	0	0	0	0	
K3	0	X	√	0	0	0	0	0	0	0	
K4	0	0	0	√	0	0	0	0	0	0	Off-Grid
K5	0	X	X	0	√	0	0	0	0	0	
K6	0	X	0	0	0	√	0	0	0	0	
K7	0	0	0	0	0	0	√	0	0	0	
K8	0	0	0	0	0	0	0	√	X	0	
K9	0	0	0	0	0	0	0	0	√	0	On Grid
K10	0	0	0	0	0	0	0	0	0	√	
SVM Cubic Kernel											
Class	K1	K2	K3	K4	K5	K6	K7	K8	K9	K10	MG Mode
K1	√	0	0	0	0	0	0	0	0	0	
K2	0	√	0	0	0	0	0	0	0	0	
K3	0	0	√	0	0	0	0	0	0	0	
K4	0	0	0	√	0	0	0	0	0	0	Off-Grid
K5	0	X	X	0	√	0	0	0	0	0	
K6	0	0	0	0	0	√	0	0	0	0	
K7	0	0	0	0	0	0	√	0	0	0	
K8	0	0	0	0	0	0	0	√	X	0	
K9	0	0	0	0	0	0	0	0	√	0	On Grid
K10	0	0	0	0	0	0	0	0	0	√	

**Table 7:** Classification results of kernel based SVM classifiers (under STC of solar).

Class	PQEs	SVM Linear		SVM Quadratic		SVM Cubic	
		Classified Instances		Classified Instances		Classified Instances	
		Correct (√)	Incorrect (X)	Correct (√)	Incorrect (X)	Correct (√)	Incorrect (X)
K1	Normal	40	0	40	0	40	0
K2	Sag	36	4	40	0	40	0
K3	Swell	28	12	37	3	40	0
K4	Harmonics	40	0	40	0	40	0
K5	Transient-1	24	16	24	16	24	16
K6	Transient-2	32	8	32	8	40	0
K7	Transient-3	40	0	40	0	40	0
K8	Transient-1	28	12	32	8	32	8
K9	Transient-2	40	0	40	0	40	0
K10	Transient-3	40	0	40	0	40	0
Overall CA		87%		%		94%	

From the prediction results of kernel based SVM classifiers (Tables 6 and 7), it is clear that the misclassification rate was higher with the linear kernel classifier for the PQEs (Swell, PQ transients 1 and 2 in islanded and transients 1in grid connected network) than Quadratic and Cubic SVM classifiers. For the Quadratic type, the misclassification rate was moderate between Linear and Cubic type classifiers. Most of the instances of PQEs (except Transients 1) were fully classified correctly with the Cubic type of classifier. Because of reduced misclassification rate with the Cubic type classifier, the classification accuracy (94%) was significantly improved as compared to the Quadratic (91.3%) and Linear (87%) type of SVM classifiers. Thus, this study proves that the cubic kernel SVM classifier offers better performance with more suitability for classification of various PQEs in the MG model of power network than other types (Quadratic and Linear).

**Fig 16:**
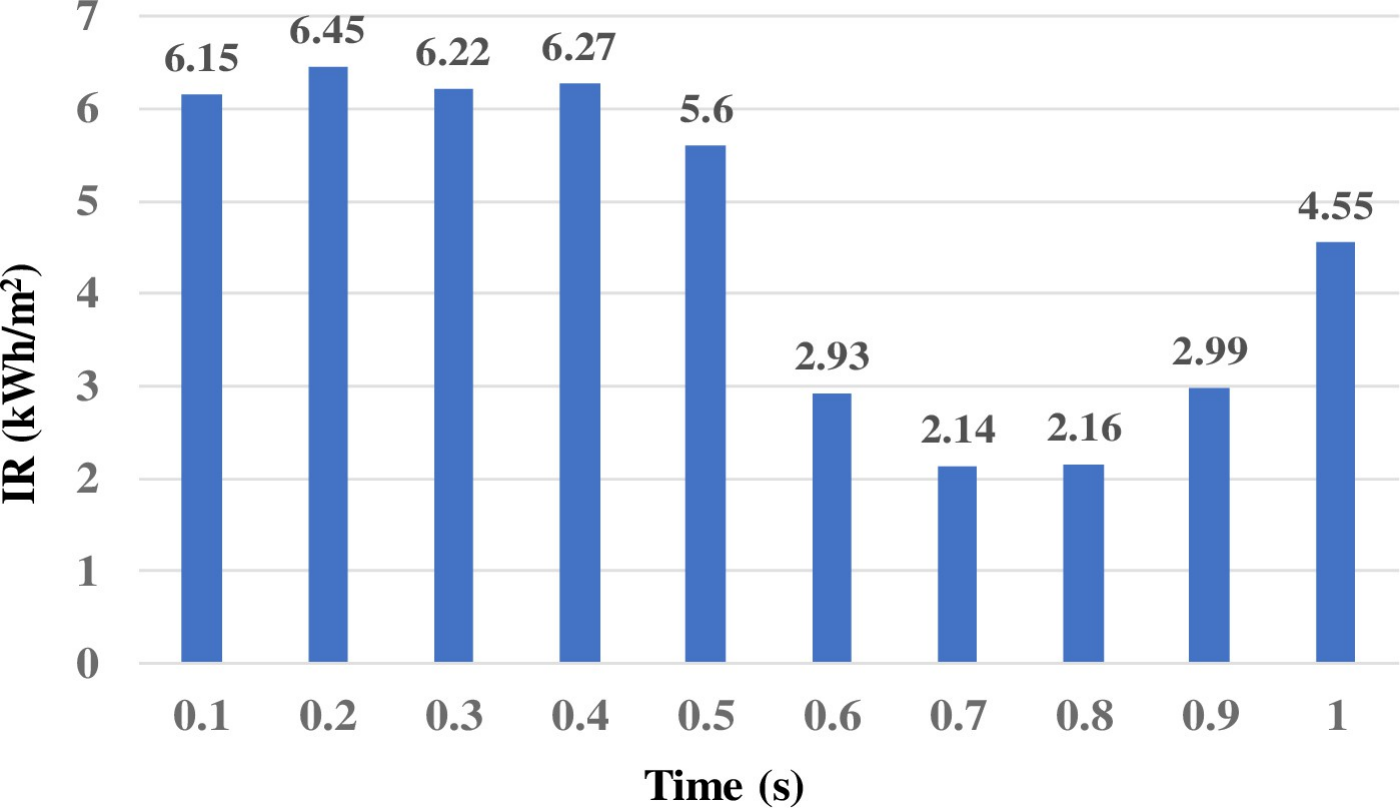
Real time varying solar data.

*(b) SVM classifiers*: *Classification results under real time varying solar*. In this analysis, the real time varying solar data for the PV source was considered as used in [37], while the analysis of all PQEs in both the on-grid and off-grid modes of the MG network. The real time solar data for the period of 1 s (with 10 slots of 0.1 s intervals) is shown in Fig 16.

Based on the Confusion Matrix results (like STC case analysis,) the classification results of Kernel based SVM classifiers were obtained, as given in Table 8. Likewise, the results of STC analysis showed that the misclassification rate was higher with the Linear kernel SVM classifier than with Quadratic and Cubic type classifiers. Also, the misclassification rate was moderate with Quadratic and significantly reduced with the Cubic type SVM classifier under this case condition. However, the results of classification accuracy were slightly lower for all the classifiers than the results obtained with STC case analysis. In comparison to the Cubic type SVM classifier, the misclassification rate of analysed PQEs was high with the Linear and Quadratic types. Thus, the cubic kernel SVM classifier provides higher classification accuracy (91.1%) than the accuracy levels of the Quadratic (88%) and Linear (83.3%) kernel SVM classifiers. This analysis clearly shows that the Cubic kernel SVM classifier provides more promising results (more than 91%) than other types, even when the PV source’s solar irradiance varies.

**Table 8:** Classification results of kernel based SVM classifiers (under varying solar at real time).

Class	PQEs	SVM Linear		SVM Quadratic		SVM Cubic	
		Classified Instances		Classified Instances		Classified Instances	
		Correct (√)	Incorrect (X)	Correct (√)	Incorrect (X)	Correct (√)	Incorrect (X)
K1	Normal	40	0	40	0	40	0
K2	Sag	40	0	40	0	40	0
K3	Swell	16	24	24	16	36	4
K4	Harmonics	40	0	40	0	40	0
K5	Transient-1	24	16	24	16	24	16
K6	Transient-2	25	15	32	8	32	8
K7	Transient-3	40	0	40	0	40	0
K8	Transient-1	28	12	32	8	33	7
K9	Transient-2	40	0	40	0	40	0
K10	Transient-3	40	0	40	0	40	0
Overall CA		83.3%		88.0%		91.3%	

Among the results of accuracy, as summarized in Table 9, it can be concluded that the classification accuracy was lower with the SVM linear kernel compared to the SVM quadratic and SVM cubic kernel classifiers. Since the SVM linear mostly provides better solutions for multi-class linear problems than non-linear, the misclassification rate was high with the non-linear nature of PQ transients, whereas the polynomial SVM kernel (quadratic and cubic) provides better solutions for non-linear PQ transients with a reduced misclassification rate. Moreover, the order of polynomial functions has a significant impact on the performance of polynomial kernel classifiers. As a result, the cubic kernel SVM classifier has a higher order of polynomial function and provides higher classification accuracy under STC and varying solar conditions than the quadratic type.

**Table 9:** Overall classification accuracy of SVM kernel classifiers under all conditions.

SVM kernel types	Accuracy (%) under STC of PV	Accuracy (%) under varying solar
SVM linear	87	83.3
SVM quadratic	91.3	88
SVM cubic	94	91.3

Furthermore, in order to improve the generalisation ability and overall accuracy of SVM classifiers, it is proposed SVM-based RS ensemble classifier for analysis. The results of classification analysis with the RS ensemble method are discussed in detail in the following section.

#### 6.2.2 Results of classification with proposed RS ensemble classifier

**Fig 17:**
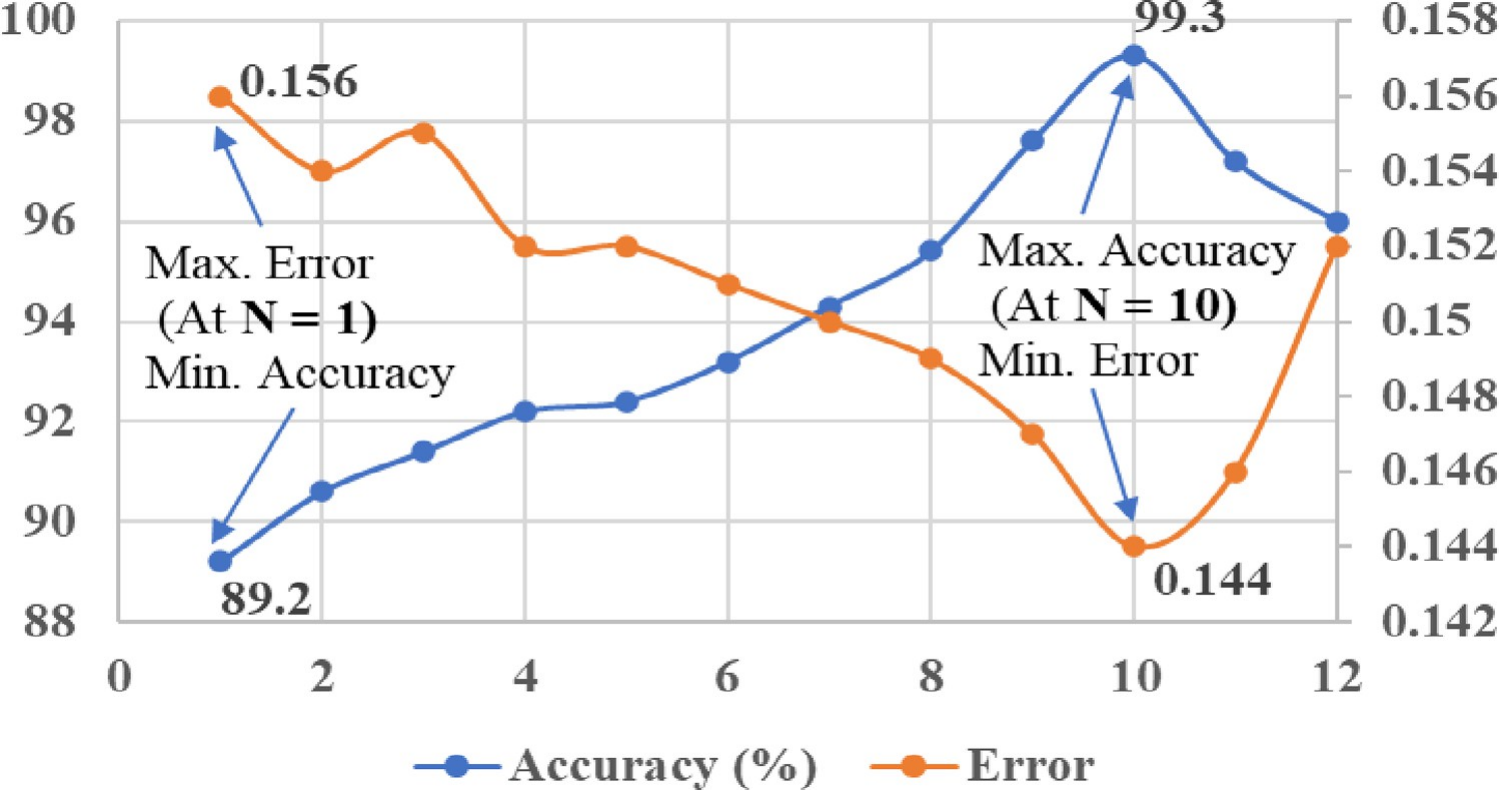
Tuning results of ensemble size with RS ensemble classifier.

In the RS ensemble method, randomly picked subsets of features (n) from the full space of the input data set (D) are used to train the N number of base classifiers, and the predictions of the base classifiers are computed by using the majority voting rule. The size of the feature subset (subspace size) and the size of the ensemble (number of base classifiers) have a significant influence over the expected converge and performance of the RS ensemble classifier [61]. Hence, it is necessary to select the appropriate value of subspace feature size and number of base classifiers (ensemble size) in the RS ensemble model. As a rule of thumb, selecting the size of the feature subset, as n = D/2 features, can yield promising results with the RS ensemble classifier [62, 63]. Therefore, in this work, feature subset size 0.5 was considered, and the optimum value of ensemble size was obtained through manual tuning. The tuning results of ensemble size with the proposed RS model, as shown in Fig 17, clearly indicates that maximum classification accuracy (99.3%) and minimum error (0.144) were achieved with ensemble size of 10 (among 1 to 12 analyses). As from the analysis results of kernel based SVM classifiers, the Cubic kernel SVM classifier was more effective and attained higher classification accuracy (94%) than other kernel types. As a result, the effective Cubic kernel SVM classifier was considered as the base classifier in the proposed RS ensemble model to achieve further improvement in its classification performance.

*(a) RS ensemble classifier*: *Classification results under STC of solar PV*. In this analysis, the classification analysis was carried out with the proposed RS ensemble model to discriminate between different PQEs in the MG network under STC of solar PV. The confusion matrix results and details of classified instances for all the PQEs in the MG network are illustrated in Tables 10 and 11, respectively. From the prediction results of the RS ensemble classifier (Tables 10 and 11), it can be noticed that all the instances of most of the PQEs (Normal, Sag, harmonics, Transients 2 and 3 with islanded, and all the transients in grid connected MG) were fully classified correctly (100%) and only one instance with PQE of Voltage Swell and two instances with Transients 1 of the islanded MG network were misclassified. Because of this higher successive classification rate, the classification accuracy (99.3%) of the RS ensemble classifier was significantly improved as compared to the Cubic kernel SVM (94%) and other kernel types (Quadratic (91.3%) and Linear (87%)). Thus, the proposed RS ensemble model is more suitable to discriminate all the PQEs in the MG network with a substantial improvement in classification accuracy compared to individual kernel based SVM classifiers.

**Table 10:** Results of confusion matrix: RS ensemble classifier.

RS Ensemble											
Class	K1	K2	K3	K4	K5	K6	K7	K8	K9	K10	MG Mode
**K1**	√	0	0	0	0	0	0	0	0	0	
**K2**	0	√	0	0	0	0	0	0	0	0	
**K3**	0	0	√	X	0	0	0	0	0	0	
**K4**	0	0	0	√	0	0	0	0	0	0	Off-Grid
**K5**	0	0	X	0	√	0	0	0	0	0	
**K6**	0	0	0	0	0	√	0	0	0	0	
**K7**	0	0	0	0	0	0	√	0	0	0	
**K8**	0	0	0	0	0	0	0	√	0	0	
**K9**	0	0	0	0	0	0	0	0	**√**	0	On Grid
**K10**	0	0	0	0	0	0	0	0	0	√	

**Table 11:** Classification results of RS ensemble classifier (under STC of solar).

Class	PQEs	RS Ensemble	
		Classified Instances	
		Correct (√)	Incorrect (X)
**K1**	Normal	40	0
**K2**	Sag	40	0
**K3**	Swell	39	1
**K4**	Harmonics	40	0
**K5**	Transient-1	38	2
**K6**	Transient-2	40	0
**K7**	Transient-3	40	0
**K8**	Transient-1	40	0
**K9**	Transient-2	40	0
**K10**	Transient-3	40	0
	Overall CA 99.3%		

*(b) RS ensemble classifier*: *Classification results under real time varying solar*. In this case of analysis, the classification analysis was carried out with the SVM based RS ensemble classifier to discriminate different PQEs in the MG network under the variation of solar PV irradiance in real time According to the results of the confusion matrix, details of classified instances for all the PQEs in the MG network and evaluated overall classification accuracy for the proposed RS ensemble classifier are illustrated in Table 12. Likewise, the classification results obtained under STC case analysis showed that the instances of most of the PQEs were fully classified correctly (100%) and only 2 instances of Swell, 6 instances of Transients 1 of an islanded network, and 4 instances of Transients 1 of a grid connected network were misclassified, respectively. As compared to the results of kernel based SVM classifiers (under both case analysis of STC and real time solar variation), the proposed ensemble classifier still provides promising results of higher classification accuracy (97%) than kernel based SVM classifiers (Cubic (94% with STC and 91.3% with varying solar)), (Quadratic (91.3% with STC and 88% with varying solar)), and (Linear (87% with STC and 83.3% with varying solar)). As compared to the classification results of the RS ensemble classifier under STC case analysis, the classification accuracy was slightly reduced under real time varying irradiance of solar PV. However, the proposed RS ensemble classifier still provides promising results with higher classification accuracy than individual kernel based SVM classifiers, even under uncertain conditions solar PV.

**Table 12:** Classification results of RS ensemble classifiers (under varying solar at real time).

Class	PQEs	RS Ensemble	
		Classified Instances	
		Correct (√)	Incorrect (X)
**K1**	Normal	40	0
**K2**	Sag	40	0
**K3**	Swell	38	2
**K4**	Harmonics	40	0
**K5**	Transient-1	34	6
**K6**	Transient-2	40	0
**K7**	Transient-3	40	0
**K8**	Transient-1	36	4
**K9**	Transient-2	40	0
**K10**	Transient-3	40	0
	Overall CA 97%		

From the summary of classification accuracy, as illustrated in Table 13, a significant improvement in accuracy was achieved with the RS ensemble method under STC and uncertain conditions of solar power. Thus, the RS ensemble classifier gains the advantages by utilising randomly selected subset features with the adoption of an ensemble strategy for the assigned set of SVM classifiers, which can reflect the classification of PQEs with higher classification accuracy and lower bias risk.

**Table 13:** Overall classification accuracy of SVM kernels and RS ensemble classifiers under all conditions.

SVM kernel types	Accuracy (%) under STC of PV	Accuracy (%) under varying solar
SVM linear	87	83.3
SVM quadratic	91.3	88
SVM cubic	94	91.3
RS ensemble	99.3	97

### 6.3 Performance analysis

In performance analysis, the Performance factors (PF) such as KS, Precision, Recall, F-Measure, and ROC of classifiers (proposed RS ensemble and kernel based SVM classifiers) were evaluated to verify the effectiveness of classifiers in further levels under STC and varying solar irradiance of PV with real time conditions.

#### 6.3.1 Results of PF under STC of PV

**Fig 18:**
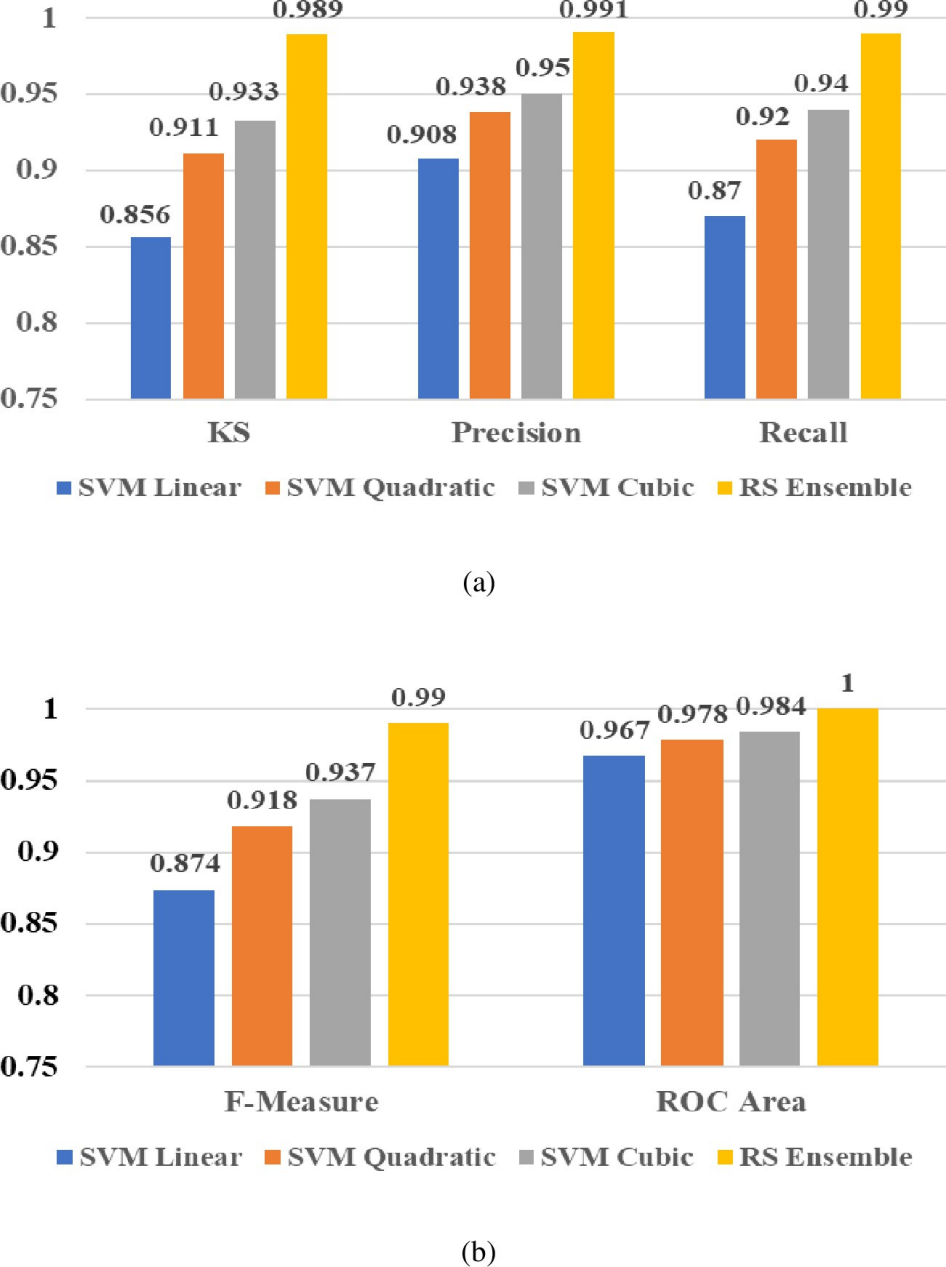
PF Results: (a) KS, Precision, and Recall; (b) F-Measure and Recall.

From the results of PF as shown in Fig 18A, it is clear that values of KS (0.989), Precision (0.991), and Recall (0.990) were significantly improved with the proposed RS ensemble classifier than the values of PF (KS, Precision, and Recall) for the kernel based SVM classifiers (Linear (0.856, 0.908, and 0.870), Quadratic (0.911, 0.938, and 0.920), and Cubic (0.933, 0.950, and 0.940)). Similarly, the results as shown in Fig 18B clearly indicate that the RS ensemble classifier offers excellent performance in terms of evaluating other PF such as F-Measure (0.975) and ROC (1.0) than the results of kernel based SVM classifiers (linear, quadratic, and Cubic). Thus, from the performance analysis, it can be concluded that the proffered RS ensemble classifier is more superior and outperforms individual kernel based SVM classifiers (linear, quadratic, and Cubic) under this condition.

#### 6.3.2 Results of PF under varying solar irradiance

Under the uncertain condition of solar PV, the results of PF obtained for all the classifiers are given in Table 14. As from the results, it can be noticed that the KS value (0.967) was high and substantially improved with the RS ensemble classifier than with kernel based SVM classifiers. Furthermore, a significant improvement in Precision (0.973), Recall (0.970), and F-Measure (0.969) results were observed with the proffered RS ensemble classifier rather than individual kernel based SVM classifiers. Likewise, the range of ROC values (from 0.961 to 0.976) with kernel based SVM classifiers was improved significantly (0.997) with the proffered RS ensemble classifier. As compared to the results of STC case analysis, the PF results were slightly reduced for all the classifiers under varying solar of PV. However, the proffered RS ensemble classifier still provides more promising results in PF than other kernel based SVM classifiers, even under uncertain conditions of solar PV.

**Table 14:** Results of PF under varying solar irradiance of PV.

Performance under real time variation of Solar				
Performance Factors	SVM Linear	SVM Quadratic	SVM Cubic	RS Ensemble
KS	0.811	0.867	0.900	0.967
Precision	0.900	0.922	0.932	0.973
Recall	0.830	0.880	0.910	0.970
F-Measure	0.833	0.883	0.909	0.969
ROC Area	0.961	0.972	0.976	0.997

The proposed RS ensemble classifier can benefit (higher accuracy with superior performance) from the use of random subspaces and the application of the ensemble strategy over the predictions of assigned Cubic kernel SVM models. Thus, the results of this study prove that the proffered RS ensemble model is more effective and robust for discriminating between different PQEs in both modes of MG network under STC and uncertain conditions of solar PV.

## 7. Comparative analysis with exiting literature works and non-linear classifiers

This section describes a comparative analysis of PQEs classification between the proposed RS ensemble method and other literature works. From the results of comparison, as illustrated in the Table 15, it is evident that the classification accuracy of different classifiers varies from the ranges of 95.30% to 100%. According to the Table 13, the research works in [14, 33] were considered to study different PQEs in simple power networks without integration of RE sources, whereas the research works in [32, 35, 64] were discriminated different PQEs in RE integrated MG networks but failed to analyse under uncertain RE source conditions. However, in this study, different PQEs and transients due to switching and LG fault events were considered to be categorised with the proposed RS ensemble method in the PV integrated MG network under real-time varying solar irradiance of the PV system. As compared to other works, the proposed RS ensemble method is more robust in discriminating PQEs with an accuracy of 97% even under uncertain conditions of the RE source. Furthermore, comparison of accuracy between the proposed RS ensemble method and other non-linear classifiers is illustrated in Table 14. From the results of the comparison (Table 16), it is inferred that the proposed method offers promising results for the classification of different PQEs in the MG network compared with other non-linear algorithms under STC and varying solar irradiance of PV in real-time conditions.

**Table 15:** Comparison of proposed method with other works.

S.NO	References	Classification techniques	Description	Accuracy (%)
1	[32]	Ensemble Bagging	Considered to analyse different PQEs in RE integrated MG network, but fails to study the effect of ensemble classifier under uncertain conditions of RE sources with real time,	95.3
2	[14]	ANN + DT	Analysed PQEs in simple power network without integration of renewable energy sources	99.9
3	[33]	Adaboost	Considered to analyse various PQEs in power distribution system without consideration of any RE sources	99.37
4	[64]	Ensemble Bagging	Considered to analyse multiple PQEs in PV integrated MG system, but fails to study with presence of PV under uncertain conditions	98
5	[35]	Ensemble voting	Analysed various PQEs in both modes of PV integrated MG network, but fails to analyse uncertain condition of PV power due to varying solar irradiance in real time condition	100
6	Proposed method	SVM based RS ensemble	Analysed various PQEs, transients due to switching events and LG fault in PV integrated MG network under real time varying solar irradiance of PV system	97

**Table 16:** Comparison of proposed method with non-linear classifiers.

S.NO	Classification techniques	Accuracy (%) under STC of PV	Accuracy (%) under varying solar
1	Multi-layer perceptron	70	67.98
2	Logistic Regression	85.76	81.43
3	J48 Decision tree	81.10	78.62
4	Proposed RS ensemble	99.3	97

## 8. Conclusions

In this study, SVM based RS ensemble classification model is proposed to detect and discriminate the most common PQEs like sag, swell, distortion of harmonics in off-grid, and different PQ transients in both the on-grid and off-grid modes of the PV integrated MG network under the following conditions: 1) STC of solar PV; and 2) varying solar irradiance of PV with real time conditions. The effectiveness of the proffered RS ensemble model is verified with the results of kernel based individual SVM classifiers (linear, polynomial (Quadratic and Cubic)). The Matlab-Simulink software tool is used to develop and simulate the PV integrated MG network for analysis. In the pre-stage of classification, the features of energy values from the disturbance signals of various PQEs are extracted by the DWT technique. Further, the input features are used to train the RS ensemble classifier and individual kernel based SVM classifiers (Linear, Quadratic, and Cubic) to obtain targeted class labels at the final stage of classification. From the classification results, it is inferred that the proffered RS ensemble classifier offers higher accuracy of classification under STC (99.3%) and varying solar condition (97%) of PV than individual kernel based SVM classifiers (Linear (87% with STC and 83.3% with solar variation), Quadratic (91.3% with STC and 88% with solar variation), and Cubic (94% with STC and 91.3% with solar variation)). Furthermore, the effectiveness of the RS ensemble classifier is verified at a further level with performance analysis. The PF results clearly show that the proffered RS ensemble model provides more promising results in PF than individual kernel based SVM classifiers. Thus, from this study, it can be concluded that the proffered SVM based RS ensemble model is more robust and offers excellent performance for classification of different PQEs in PV connected MG network under STC and uncertain conditions of solar PV. Furthermore, classification of complex PQEs using hybrid signal processing method with advanced intelligent classifiers in the MG power network is the future scope of this work.

## Supporting information

S1 File (DOCX)
