# Supplementary material for: A random subspace ensemble classification model for discrimination of power quality events in solar PV microgrid power network
Source: PLoS One. 2022 Jan 27;17(1):e0262570. doi: 10.1371/journal.pone.0262570 (PMC8794120; doi:10.1371/journal.pone.0262570)
Supplement: S1 File — (DOCX) [file pone.0262570.s001.docx]

Data for Classification:

**On-Grid Mode** : The data features for various power quality events during on- grid mode is presented as follows:

| **Classes** | | **Features** | | |
| --- | --- | --- | --- | --- |
|  |  | **Energy-A** | **Energy-B** | **Energy-C** |
| K1 | Normal | 592.91 | 251.32 | 646.92 |
| K1 | Normal | 592.62 | 251.3 | 646.9 |
| K1 | Normal | 592.54 | 251.28 | 646.88 |
| K1 | Normal | 592.72 | 251.34 | 646.94 |
| K1 | Normal | 592.88 | 251.33 | 646.82 |
| K1 | Normal | 592.85 | 251.35 | 646.86 |
| K1 | Normal | 592.83 | 251.32 | 646.82 |
| K1 | Normal | 592.78 | 251.24 | 646.78 |
| K1 | Normal | 592.74 | 251.21 | 646.74 |
| K1 | Normal | 592.77 | 251.19 | 646.7 |
| K1 | Normal | 592.68 | 251.17 | 646.68 |
| K1 | Normal | 592.69 | 251.14 | 646.65 |
| K1 | Normal | 592.64 | 251.12 | 646.62 |
| K1 | Normal | 592.59 | 251.11 | 646.59 |
| K1 | Normal | 592.58 | 251.07 | 646.58 |
| K1 | Normal | 592.48 | 251.05 | 646.54 |
| K1 | Normal | 592.42 | 251.03 | 646.52 |
| K1 | Normal | 592.39 | 250.96 | 646.49 |
| K1 | Normal | 592.36 | 250.93 | 646.46 |
| K1 | Normal | 592.32 | 250.9 | 646.43 |
| K2 | SAG | 753.19 | 402.03 | 358.35 |
| K2 | SAG | 753.61 | 402.94 | 357.62 |
| K2 | SAG | 749.52 | 423.02 | 340.13 |
| K2 | SAG | 749.6 | 423.21 | 340.22 |
| K2 | SAG | 745.83 | 443.15 | 326.54 |
| K2 | SAG | 746.82 | 442.52 | 325.96 |
| K2 | SAG | 743.26 | 461.56 | 313.63 |
| K2 | SAG | 741.55 | 461.52 | 314.46 |
| K2 | SAG | 736.07 | 476.22 | 304.14 |
| K2 | SAG | 736.01 | 477.45 | 304.75 |
| K2 | SAG | 734.22 | 479.21 | 294.52 |
| K2 | SAG | 732.43 | 478.88 | 294.62 |
| K2 | SAG | 732.07 | 479.15 | 294.14 |
| K2 | SAG | 732.01 | 479.52 | 294.75 |
| K2 | SAG | 731.92 | 479.56 | 294.12 |
| K2 | SAG | 731.83 | 480.12 | 291.62 |
| K2 | SAG | 731.77 | 480.22 | 290.14 |
| K2 | SAG | 731.69 | 480.45 | 289.75 |
| K2 | SAG | 731.62 | 481.21 | 284.52 |
| K2 | SAG | 731.73 | 481.88 | 282.62 |
| K3 | SWELL | 714.07 | 330.84 | 392.46 |
| K3 | SWELL | 714.63 | 330.31 | 392.26 |
| K3 | SWELL | 712.93 | 369.4 | 351.15 |
| K3 | SWELL | 712.63 | 369.56 | 350.26 |
| K3 | SWELL | 706.59 | 419.01 | 308.58 |
| K3 | SWELL | 705.54 | 419.39 | 308.58 |
| K3 | SWELL | 684.61 | 472.69 | 274.66 |
| K3 | SWELL | 684.88 | 477.86 | 274.72 |
| K3 | SWELL | 650.38 | 529.22 | 252.1 |
| K3 | SWELL | 650.8 | 528.64 | 251.99 |
| K3 | SWELL | 608.08 | 581.06 | 242.15 |
| K3 | SWELL | 606.5 | 581.59 | 241.85 |
| K3 | SWELL | 558.18 | 629.4 | 245.77 |
| K3 | SWELL | 557.2 | 628.46 | 245.88 |
| K3 | SWELL | 554.18 | 629.4 | 246.77 |
| K3 | SWELL | 551.2 | 632.46 | 247.88 |
| K3 | SWELL | 548.18 | 631.4 | 247.77 |
| K3 | SWELL | 546.2 | 638.46 | 248.88 |
| K3 | SWELL | 538.18 | 639.4 | 249.77 |
| K3 | SWELL | 537.2 | 641.46 | 249.88 |
| K4 | Harmonics | 358.6 | 295.47 | 135.6 |
| K4 | Harmonics | 358.05 | 295.19 | 135.51 |
| K4 | Harmonics | 353.44 | 282.45 | 135.99 |
| K4 | Harmonics | 353.68 | 282.91 | 135.73 |
| K4 | Harmonics | 349.54 | 265.37 | 136.9 |
| K4 | Harmonics | 348.63 | 265.62 | 136.52 |
| K4 | Harmonics | 345.76 | 243.45 | 139.26 |
| K4 | Harmonics | 345.9 | 243.62 | 138.88 |
| K4 | Harmonics | 342.03 | 215.13 | 144.5 |
| K4 | Harmonics | 341.38 | 215.16 | 144.75 |
| K4 | Harmonics | 338.31 | 178.93 | 148.36 |
| K4 | Harmonics | 334.34 | 178.83 | 149.03 |
| K4 | Harmonics | 331.31 | 178.93 | 152.36 |
| K4 | Harmonics | 328.34 | 178.83 | 153.13 |
| K4 | Harmonics | 326.31 | 168.03 | 155.03 |
| K4 | Harmonics | 324.34 | 165.82 | 158.18 |
| K4 | Harmonics | 321.31 | 160.12 | 163.53 |
| K4 | Harmonics | 318.34 | 162.82 | 159.13 |
| K4 | Harmonics | 315.31 | 161.82 | 162.29 |
| K4 | Harmonics | 314.34 | 167.82 | 164.03 |
| K5 | Transient1_ PV Inverter Switching | 738.62 | 490.54 | 365.11 |
| K5 | Transient1_ PV Inverter Switching | 729.31 | 426.07 | 320.87 |
| K5 | Transient1_ PV Inverter Switching | 727.93 | 414.64 | 312.25 |
| K5 | Transient1_ PV Inverter Switching | 703.42 | 386.14 | 279.53 |
| K5 | Transient1_ PV Inverter Switching | 698.22 | 380.22 | 269.22 |
| K5 | Transient1_ PV Inverter Switching | 678.12 | 376.12 | 259.58 |
| K5 | Transient1_ PV Inverter Switching | 670.12 | 375.42 | 238.22 |
| K5 | Transient1_ PV Inverter Switching | 668.12 | 365.33 | 220.46 |
| K5 | Transient1_ PV Inverter Switching | 658.44 | 360.23 | 209.98 |
| K5 | Transient1_ PV Inverter Switching | 654.34 | 289.22 | 198.98 |
| K5 | Transient1_ PV Inverter Switching | 652.24 | 278.19 | 212.43 |
| K5 | Transient1_ PV Inverter Switching | 650.32 | 279.21 | 234.42 |
| K5 | Transient1_ PV Inverter Switching | 648.44 | 272.23 | 236.72 |
| K5 | Transient1_ PV Inverter Switching | 644.14 | 269.12 | 239.12 |
| K5 | Transient1_ PV Inverter Switching | 638.31 | 268.23 | 242.56 |
| K5 | Transient1_ PV Inverter Switching | 634.34 | 265.26 | 272.54 |
| K5 | Transient1_ PV Inverter Switching | 632.12 | 263.43 | 267.12 |
| K5 | Transient1_ PV Inverter Switching | 630.32 | 260.23 | 289.13 |
| K5 | Transient1_ PV Inverter Switching | 628.44 | 267.23 | 299.44 |
| K5 | Transient1_ PV Inverter Switching | 624.34 | 269.83 | 298.12 |
| K6 | Transient2_Capacitor Switching | 813.72 | 444.32 | 375.25 |
| K6 | Transient2_Capacitor Switching | 813.44 | 443.35 | 375.82 |
| K6 | Transient2_Capacitor Switching | 802.31 | 585.13 | 312.24 |
| K6 | Transient2_Capacitor Switching | 802.39 | 584.96 | 312.58 |
| K6 | Transient2_Capacitor Switching | 797.97 | 740.11 | 295.17 |
| K6 | Transient2_Capacitor Switching | 788.15 | 740.06 | 294.94 |
| K6 | Transient2_Capacitor Switching | 681.65 | 881.48 | 346.95 |
| K6 | Transient2_Capacitor Switching | 671.22 | 883.24 | 347.5 |
| K6 | Transient2_Capacitor Switching | 630.76 | 997.22 | 368.92 |
| K6 | Transient2_Capacitor Switching | 632.23 | 995.23 | 369.56 |
| K6 | Transient2_Capacitor Switching | 623.72 | 444.32 | 375.25 |
| K6 | Transient2_Capacitor Switching | 613.44 | 443.35 | 375.82 |
| K6 | Transient2_Capacitor Switching | 622.31 | 585.13 | 312.24 |
| K6 | Transient2_Capacitor Switching | 602.39 | 584.96 | 312.58 |
| K6 | Transient2_Capacitor Switching | 637.97 | 740.11 | 295.17 |
| K6 | Transient2_Capacitor Switching | 688.15 | 740.06 | 294.94 |
| K6 | Transient2_Capacitor Switching | 671.65 | 881.48 | 346.95 |
| K6 | Transient2_Capacitor Switching | 681.22 | 883.24 | 347.5 |
| K6 | Transient2_Capacitor Switching | 660.76 | 997.22 | 368.92 |
| K6 | Transient2_Capacitor Switching | 662.23 | 995.23 | 369.56 |
| K7 | Transients3_LG Fault | 1456.22 | 19.4 | 1290 |
| K7 | Transients3_LG Fault | 1460.34 | 26.56 | 1168 |
| K7 | Transients3_LG Fault | 1455 | 41.37 | 1056 |
| K7 | Transients3_LG Fault | 1411 | 62.5 | 987.73 |
| K7 | Transients3_LG Fault | 1398.22 | 66.23 | 978.12 |
| K7 | Transients3_LG Fault | 1392.12 | 66.34 | 971.23 |
| K7 | Transients3_LG Fault | 1378.21 | 69.21 | 968.12 |
| K7 | Transients3_LG Fault | 1218.62 | 74.34 | 958.22 |
| K7 | Transients3_LG Fault | 1255.21 | 78.53 | 898.43 |
| K7 | Transients3_LG Fault | 1151.24 | 80.46 | 666.69 |
| K7 | Transients3_LG Fault | 1178.22 | 82.44 | 634.21 |
| K7 | Transients3_LG Fault | 1159.34 | 85.62 | 622.18 |
| K7 | Transients3_LG Fault | 990.13 | 87.31 | 592.17 |
| K7 | Transients3_LG Fault | 998.65 | 91.34 | 569.72 |
| K7 | Transients3_LG Fault | 986.54 | 91.45 | 522.12 |
| K7 | Transients3_LG Fault | 982.23 | 92.13 | 518.92 |
| K7 | Transients3_LG Fault | 978.12 | 92.34 | 498.68 |
| K7 | Transients3_LG Fault | 971.42 | 92.89 | 488.72 |
| K7 | Transients3_LG Fault | 960.22 | 93.22 | 469.66 |
| K7 | Transients3_LG Fault | 958.42 | 94.52 | 469.9 |

**OFF-Grid Mode** : The data features for various power quality events during off- grid mode is presented as follows:

| **Classes** | | **Features** | | |
| --- | --- | --- | --- | --- |
|  |  | **Energy-A** | **Energy-B** | **Energy-C** |
| K8 | Transients1_PV Inverter Switching | 534.12 | 695.44 | 270.02 |
| K8 | Transients1_PV Inverter Switching | 534.16 | 695.39 | 270.33 |
| K8 | Transients1_PV Inverter Switching | 534.22 | 695.62 | 271.13 |
| K8 | Transients1_PV Inverter Switching | 533.18 | 695.69 | 271.46 |
| K8 | Transients1_PV Inverter Switching | 533.54 | 695.75 | 269.85 |
| K8 | Transients1_PV Inverter Switching | 534.16 | 695.92 | 269.82 |
| K8 | Transients1_PV Inverter Switching | 534.37 | 695.54 | 269.84 |
| K8 | Transients1_PV Inverter Switching | 534.26 | 695.94 | 270.32 |
| K8 | Transients1_PV Inverter Switching | 533.28 | 696.12 | 270.12 |
| K8 | Transients1_PV Inverter Switching | 533.98 | 696.23 | 270.22 |
| K8 | Transients1_PV Inverter Switching | 532.17 | 696.32 | 270.43 |
| K8 | Transients1_PV Inverter Switching | 533.29 | 695.49 | 269.92 |
| K8 | Transients1_PV Inverter Switching | 533.75 | 695.52 | 269.72 |
| K8 | Transients1_PV Inverter Switching | 533.19 | 695.57 | 269.66 |
| K8 | Transients1_PV Inverter Switching | 533.67 | 696.26 | 269.9 |
| K8 | Transients1_PV Inverter Switching | 533.29 | 695.47 | 269.91 |
| K8 | Transients1_PV Inverter Switching | 532.12 | 694.88 | 269.76 |
| K8 | Transients1_PV Inverter Switching | 531.14 | 694.76 | 269.52 |
| K8 | Transients1_PV Inverter Switching | 531.72 | 695.16 | 269.57 |
| K8 | Transients1_PV Inverter Switching | 531.62 | 695.34 | 268.42 |
| K9 | Transients2_Capacitor Switching | 535.93 | 696.74 | 270.33 |
| K9 | Transients2_Capacitor Switching | 535.2 | 696.56 | 271.05 |
| K9 | Transients2_Capacitor Switching | 535.36 | 695.98 | 270.48 |
| K9 | Transients2_Capacitor Switching | 535.12 | 696.77 | 270.83 |
| K9 | Transients2_Capacitor Switching | 535.89 | 697.18 | 270.54 |
| K9 | Transients2_Capacitor Switching | 535.37 | 696.79 | 269.93 |
| K9 | Transients2_Capacitor Switching | 535.6 | 696.98 | 270.12 |
| K9 | Transients2_Capacitor Switching | 535.82 | 697.15 | 270.32 |
| K9 | Transients2_Capacitor Switching | 535.57 | 696.81 | 270.12 |
| K9 | Transients2_Capacitor Switching | 535.32 | 696.7 | 269.88 |
| K9 | Transients2_Capacitor Switching | 535.16 | 696.52 | 269.22 |
| K9 | Transients2_Capacitor Switching | 535.76 | 698.22 | 271.22 |
| K9 | Transients2_Capacitor Switching | 535.94 | 698.71 | 270.82 |
| K9 | Transients2_Capacitor Switching | 535.87 | 698.43 | 269.48 |
| K9 | Transients2_Capacitor Switching | 535.98 | 697.54 | 270.99 |
| K9 | Transients2_Capacitor Switching | 536.12 | 697.33 | 269.97 |
| K9 | Transients2_Capacitor Switching | 536.22 | 698.12 | 269.87 |
| K9 | Transients2_Capacitor Switching | 536.42 | 696.76 | 269.62 |
| K9 | Transients2_Capacitor Switching | 536.98 | 697.44 | 270.32 |
| K9 | Transients2_Capacitor Switching | 536.78 | 697.24 | 270.12 |
| K10 | Transients3_LG Fault | 1001.12 | 244.7 | 86.39 |
| K10 | Transients3_LG Fault | 987.82 | 289.08 | 111.12 |
| K10 | Transients3_LG Fault | 966.65 | 293.44 | 114.54 |
| K10 | Transients3_LG Fault | 973.23 | 294.23 | 166.87 |
| K10 | Transients3_LG Fault | 919.58 | 297.19 | 123.42 |
| K10 | Transients3_LG Fault | 908.43 | 306.38 | 127.92 |
| K10 | Transients3_LG Fault | 907.92 | 311.21 | 126.54 |
| K10 | Transients3_LG Fault | 904.32 | 312.45 | 127.72 |
| K10 | Transients3_LG Fault | 902.2 | 316.11 | 128.73 |
| K10 | Transients3_LG Fault | 900.92 | 318.55 | 132.72 |
| K10 | Transients3_LG Fault | 899.92 | 319.23 | 132.43 |
| K10 | Transients3_LG Fault | 897.54 | 320.43 | 133.21 |
| K10 | Transients3_LG Fault | 892.45 | 320.98 | 133.46 |
| K10 | Transients3_LG Fault | 894.56 | 321.56 | 133.56 |
| K10 | Transients3_LG Fault | 895.76 | 321.45 | 134.32 |
| K10 | Transients3_LG Fault | 890.13 | 324.22 | 136.12 |
| K10 | Transients3_LG Fault | 887.45 | 325.62 | 137.12 |
| K10 | Transients3_LG Fault | 886.34 | 326.72 | 137.32 |
| K10 | Transients3_LG Fault | 888.78 | 328.54 | 137.56 |
| K10 | Transients3_LG Fault | 886.84 | 332.21 | 138.43 |
